# CLU (clusterin) and PPARGC1A/PGC1α coordinately control mitophagy and mitochondrial biogenesis for oral cancer cell survival

**DOI:** 10.1080/15548627.2024.2309904

**Published:** 2024-03-06

**Authors:** Prakash P. Praharaj, Srimanta Patra, Amruta Singh, Debasna P. Panigrahi, Hwa Y. Lee, Mohammad F. Kabir, Muhammad K. Hossain, Samir K. Patra, Birija S. Patro, Shankargouda Patil, Daniel J. Klionsky, Han J. Chae, Sujit K. Bhutia

**Affiliations:** a Cancer and Cell Death Laboratory, Department of Life Science, National Institute of Technology Rourkela, Rourkela, Odisha, India; b Department of Pharmacology, Jeonbuk National University Medical School, Jeonju, Jeonbuk, Republic of Korea; c Department of Pharmacology, School of Medicine, Institute of New Drug Development, Jeonbuk National University, Jeonju, Republic of Korea; d School of Pharmacy, Jeonbuk National University, Jeonju, Jeonbuk, Republic of Korea; e Laboratory of epigenetics, Department of Life Science, National Institute of Technology Rourkela, Rourkela, Odisha, India; f Bio-Organic Division, Bhabha Atomic Research Centre, Mumbai, Maharashtra, India; g College of Dental Medicine, Roseman University of Health Sciences, South Jordan, UT, USA; h Life Sciences Institute and Department of Molecular, Cellular and Developmental Biology, University of Michigan, Ann Arbor, MI, USA; i Non-Clinical Evaluation Center, Biomedical Research Institute, Jeonbuk National University Hospital, Jeonju, Jeonbuk, Republic of Korea

**Keywords:** Clusterin, mitochondrial biogenesis, mitophagy, mitophagy-associated cell death, PPARGC1A/PGC1α

## Abstract

Mitophagy involves the selective elimination of defective mitochondria during chemotherapeutic stress to maintain mitochondrial homeostasis and sustain cancer growth. Here, we showed that CLU (clusterin) is localized to mitochondria to induce mitophagy controlling mitochondrial damage in oral cancer cells. Moreover, overexpression and knockdown of CLU establish its mitophagy-specific role, where CLU acts as an adaptor protein that coordinately interacts with BAX and LC3 recruiting autophagic machinery around damaged mitochondria in response to cisplatin treatment. Interestingly, CLU triggers class III phosphatidylinositol 3-kinase (PtdIns3K) activity around damaged mitochondria, and inhibition of mitophagic flux causes the accumulation of excessive mitophagosomes resulting in reactive oxygen species (ROS)-dependent apoptosis during cisplatin treatment in oral cancer cells. In parallel, we determined that PPARGC1A/PGC1α (PPARG coactivator 1 alpha) activates mitochondrial biogenesis during CLU-induced mitophagy to maintain the mitochondrial pool. Intriguingly, PPARGC1A inhibition through small interfering RNA (si*PPARGC1A*) and pharmacological inhibitor (SR-18292) treatment counteracts CLU-dependent cytoprotection leading to mitophagy-associated cell death. Furthermore, co-treatment of SR-18292 with cisplatin synergistically suppresses tumor growth in oral cancer xenograft models. In conclusion, CLU and PPARGC1A are essential for sustained cancer cell growth by activating mitophagy and mitochondrial biogenesis, respectively, and their inhibition could provide better therapeutic benefits against oral cancer.

## Introduction

A head and neck cancer type, oral squamous cell carcinoma (OSCC), is considered one of the six most common cancers affecting the global population [1]. At present, chemoresistance to the existing chemotherapies adversely affects the treatment outcomes, leading to a higher mortality rate among cancer patients [2,3]. Mitophagy is a type of selective autophagy where cancer cells eliminate defective mitochondria either through activation of the PINK1-PRKN/parkin pathway or through recruitment/exposure of mitophagy receptors on mitochondria to facilitate lysosomal-based degradation [4,5]. In contrast, mitochondrial biogenesis is a complex multifactorial process necessary for the genesis of new mitochondria through the coordinated action of both nuclear DNA and mitochondrial DNA (mtDNA) in response to alterations in fuel-substrates balance or bioenergetic need [6]. Moreover, mitophagy opposes tumor initiation, whereas as the tumor progresses, it acts as a protective mechanism supporting tumor growth and survival [7]. Similarly, mitochondrial biogenesis either supports or opposes tumor growth depending upon tumor stage, tissue type, or stress associated with the tumor microenvironment [8,9]. Cancer cells maintain a dynamic equilibrium between selective mitochondrial turnover by autophagy (mitophagy) and biogenesis, keeping the mitochondrial network fully functional for sustained cancer growth [10]. In this perspective, targeted inhibition of these mitochondria-centric processes such as mitophagy and mitochondrial biogenesis could provide better therapeutic benefits against oral cancer.

CLU (clusterin) is a stress-induced molecular chaperone [11], highly expressed in aggressive cancer types, regulating multiple aspects of tumor development such as growth, metastasis, epithelial-mesenchymal transition, inflammation and survival pathways [12]. CLU also protects cancer cells from apoptosis through activating class I phosphoinositide 3-kinase/PI3K-AKT pathways [13,14], and HSPA5/GRP78 activation during ER stress [15]. Moreover, CLU prevents BAX-dependent release of CYCS (cytochrome c, somatic) into the cytoplasm, which inhibits the activation of downstream caspases to attenuate apoptosis [16]. Growing evidence also reports the unique function of CLU in promoting cancer cell survival through activating an autophagy cascade, where CLU interacts with LC3 through its putative LC3-interacting region (LIR) motif, facilitating a stable LC3-ATG3 hetero-complex leading to autophagosome biogenesis [17]. CLU deficiency attenuates autophagy, preventing growth and activating apoptosis in hepatocellular carcinoma [18]. Further, we also reported that CLU-mediated autophagy provides cytoprotection against serum starvation-induced apoptosis through modulating the AMPK-MTOR-ULK1 axis in OSCC cells [19].

PPARGC1A is a transcriptional coactivator for PPARG/PPARγ (peroxisome proliferator activated receptor gamma) and is the master regulator of mitochondrial biogenesis. PPARGC1A is present mainly in the cytoplasm. However, post-translational modification such as phosphorylation and/or deacetylation promotes translocation to the nucleus, enhancing mitochondrial biogenesis [20,21]. Higher PPARGC1A activity triggers melanocyte inducing transcription factor expression, promoting a melanoma subtype [22–24]; PPARGC1A protects cancer cells from ROS-induced apoptosis, supporting cancer growth and progression in melanomas [23]. More importantly, our understanding of the molecular mechanism(s) responsible for coordinated regulation of mitochondrial autophagy and biogenesis in response to chemotherapeutic stress is scarce. Therefore, we examined whether targeting CLU and PPARGC1A could alter the mitochondrial-homeostatic machinery for better therapeutic outcomes.

The current study explored a novel function of CLU in regulating mitochondrial turnover (mitophagy) through molecular interaction with conformation-altered BAX and LC3, recruiting autophagic machinery around dysfunctional mitochondria. Coordinately, PPARGC1A activates mitochondrial biogenesis to maintain mitochondrial quality control in response to chemotherapeutic stress. Pharmacological inhibition of PPARGC1A combined with cisplatin disturbed the balance between mitophagy and mitochondrial biogenesis, leading to excessive mitophagy, showing a potent synergistic effect in *in vitro* and *in vivo* models of oral cancer.

## Results

### CLU localizes to mitochondria and protects mitochondria from damage due to cisplatin in oral cancer cells

To investigate the role of cisplatin in mitochondrial functionality, we first treated two oral cancer cell lines, CAL-33 and FaDu, with various concentrations of cisplatin (1, 5, and 10 µM) for different time points (6, 12, and 24 h) and performed flow cytometry-based mitochondrial health analysis using two mitochondria-specific dyes MitoTracker Green FM (MTG) and MitoTracker Red CMXROS (MTR). MTG stains the entire mitochondrial population irrespective of the membrane potential (ΔΨm), whereas MTR only labels functional mitochondria [25,26]. A higher accumulation of dysfunctional mitochondria (MTG-positive, MTR-negative) and a substantial decline in functional mitochondria (MTG-positive, MTR-positive) was observed with an increased concentration of cisplatin in both FaDu and CAL-33 cells (Figure S1A_i-ii_). Similarly, the time-course analysis also showed a gradual increment of dysfunctional mitochondria in cisplatin-treated groups (Figure S1B_i-ii_).

**Figure 1.**
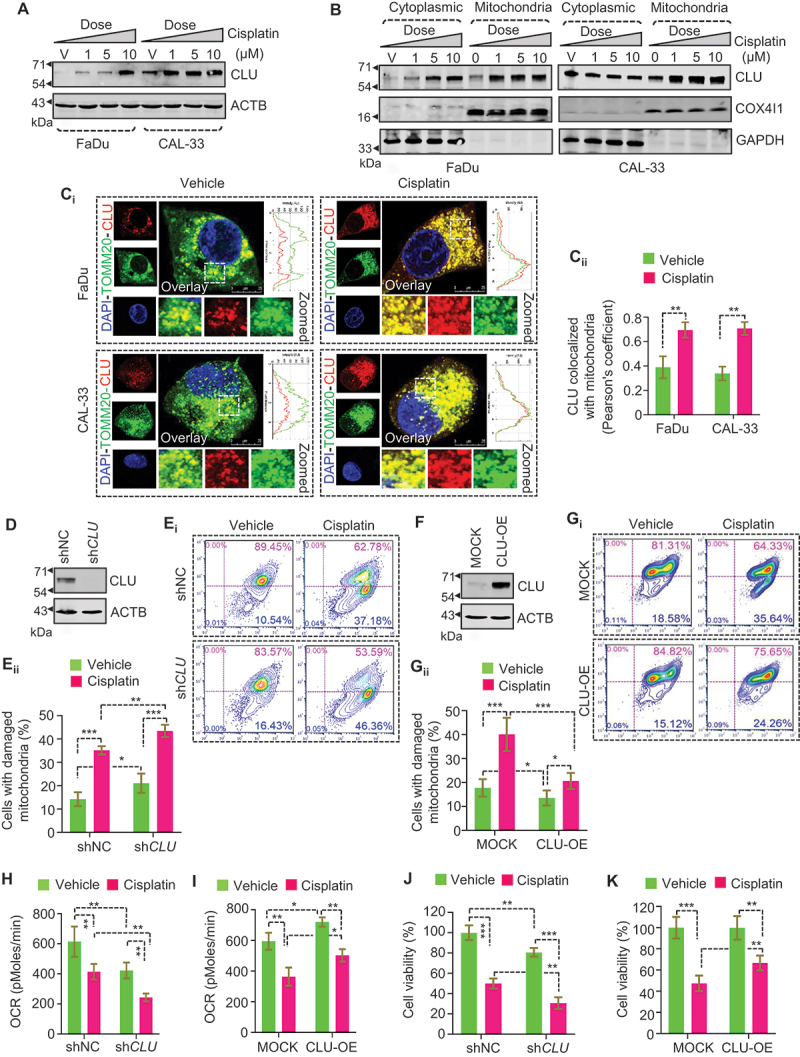
CLU maintains mitochondrial functionality through its mitochondrial translocation during cisplatin-induced membrane depolarization. (A-D) FaDu and CAL-33 cells were treated with different concentrations of cisplatin (1, 5, and 10 µM), followed by (A) western blot analysis to monitor CLU expression, (B) fractionation analysis to determine the subcellular distribution of CLU. (C_i_) Representative confocal microscopy images of FaDu and CAL-33 cells treated with cisplatin (10 µM; 24 h) followed by immunofluorescence analysis monitoring CLU (red) and TOMM20 (green). DAPI (blue) is used to stain the nucleus. Scale bars: 25 µm. (C_ii_) Quantification of colocalization (%) represented as Pearson’s co-efficient value calculated from images of each condition of three independent experiments using the JACoP plugin of ImageJ. (D, F) Western blot analysis for CLU expression in CLU overexpressed and knockdown cells (E_i_, G_i_) flow cytometry analysis using MTG and MTR shows mitochondrial damage in (E_i_) CLU-OE and (G_i_) CLU KD cells treated with cisplatin (10 µM; 24 h). (E_ii_, G_ii_) Quantification shows the cells with damaged mitochondria (%) in CLU KD and CLU-OE cells treated with cisplatin. (H-I) OCR analysis in CLU KD and CLU-OE cells treated with cisplatin (10 µM; 24 h). (J-K) MTT assay representing cell viability in CLU KD and CLU-OE cells treated with cisplatin. Data were normalized to the untreated groups or shNC or MOCK cells (mean ± S.D., *n* = 3). *p < 0.05, **p < 0.01, and ***p < 0.001.

Next, we assessed cellular oxygen consumption rate (OCR) status in both FaDu and CAL-33 cells during cisplatin treatment and noticed that the OCR was reduced in a dose-dependent manner (Figure S1C). More importantly, cisplatin treatment triggered CLU expression, and its mitochondrial distribution (mitochondrial fraction) was increased in a dose-dependent manner in both FaDu and CAL-33 cells (Figure 1A-B, S1D-E). The purity of the respective fractions was validated using specific markers such as GAPDH (cytoplasmic fraction) and COX4I1 (cytochrome c oxidase subunit 4I1; a mitochondrial inner membrane protein, mitochondrial fraction). Confocal microscopy-based colocalization analysis also showed a stronger colocalization between CLU and mitochondria (TOMM20 [translocase of outer mitochondrial membrane 20], a mitochondrial outer membrane protein) in the cisplatin-treated cells relative to the untreated ones, suggesting that CLU accumulated more obviously on mitochondria in response to chemotherapeutic stress (Figure 1C).

We extended our analysis by examining the role of endogenous CLU in cisplatin-induced mitochondrial damage, where we knocked down CLU using small hairpin RNA (sh*CLU*) in CAL-33 cells (higher basal level endogenous CLU). The knockdown efficiency of sh*CLU* was verified through marked downregulation in the expression of endogenous CLU (Figure 1D, S1F). CLU KD significantly impaired mitochondrial health as indicated by a higher accumulation of dysfunctional mitochondria, which further increased during cisplatin treatment (Figure 1E). Next, we stably expressed constructs encoding CLU or vector alone (MOCK) in FaDu cells (lower basal level endogenous CLU). The stable expression of CLU was verified through western blot analysis (Figure 1F, S1G). CLU overexpression decreased the accumulation of dysfunctional mitochondria in both untreated and cisplatin-treated conditions compared to MOCK cells (Figure 1G), suggesting the repair of mitochondrial damage. Similarly, the gain and loss of function of CLU also altered OCR levels in oral cancer cells during cisplatin treatment (Figure 1H-I). More importantly, CLU KD enhanced cisplatin-induced cytotoxicity as indicated by lower cell viability, which was reversed in CLU-overexpressing oral cancer cells during cisplatin treatment (Figure 1J-K). Collectively, our finding suggests that cisplatin promoted CLU accumulation on mitochondria to maintain a functional mitochondrial pool in oral cancer.

### CLU eliminates cisplatin-driven dysfunctional mitochondria through mitochondrial autophagy in oral squamous cell carcinoma

**Figure 2.**
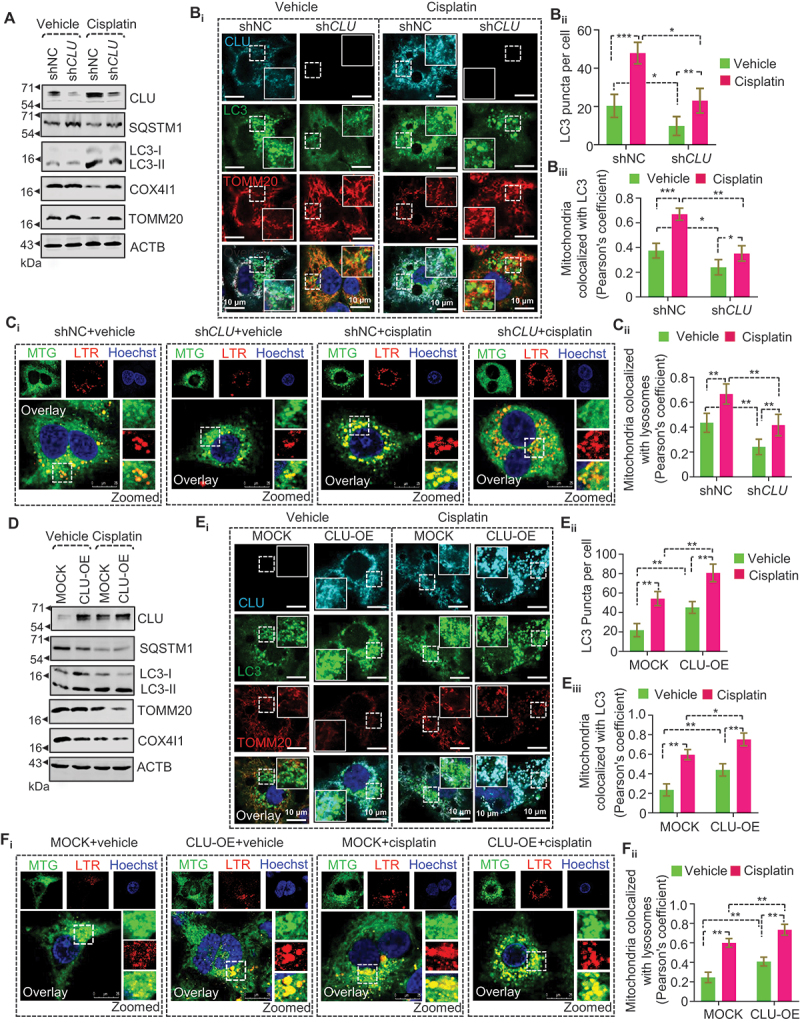
CLU regulates mitophagy status to eliminate cisplatin-induced damaged mitochondria in oral cancer cells. (A-C) CAL-33 cells expressing shNC and sh*CLU* treated with cisplatin followed by western blotting analysis showing (A) the change in expression of SQSTM1, TOMM20, COX4I1, and LC3 for mitophagy status evaluation, (B_i_) confocal microscopy-based colocalization analysis for CLU (cyan), LC3 (green; representing autophagosomes) and TOMM20 (red; representing mitochondria), and (C_i_) for LysoTracker red (LTR; representing lysosomes) and MitoTracker green (MTG; representing mitochondria). (B_ii_) Quantification showing the number of LC3 puncta per cell in shNC or sh*CLU* cells treated with cisplatin (10 µM; 24 h) and (B_iii_, C_ii_) quantification showing colocalization (%) defined as Pearson’s co-efficient value calculated from images of each condition of three independent experiments using the JACoP plugin of ImageJ. (D-F) FaDu cells stably expressing a construct encoding CLU and MOCK (empty vector) and treated with cisplatin followed by western blotting analysis showing (D) the change in expression of SQSTM1, TOMM20, COX4I1, and LC3 for mitophagy status evaluation, (E_i_) confocal microscopy-based colocalization analysis for CLU (cyan), LC3 (green; representing autophagosomes) and TOMM20 (red; representing mitochondria), and (F_i_) for LysoTracker Red (LTR; representing lysosomes) and MitoTracker Green (MTG; representing mitochondria). (E_ii_) Quantification showing the number of LC3 puncta per cells in MOCK or CLU-OE cells treated with cisplatin (10 µM; 24 h) and (E_iii_, F_ii_) quantification of colocalization (%) defined as Pearson’s co-efficient value calculated from images of each condition of three independent experiments using the JACoP plugin of ImageJ. Data were normalized to the shNC or MOCK cells or DMSO (0.1%)-treated cells (mean ± S.D., *n* = 5). *p < 0.05, and **p < 0.01.

As cisplatin treatment triggers mitochondrial dysfunction and CLU localization to mitochondria, we next examined whether CLU could activate mitophagy to eliminate dysfunctional mitochondria generated in response to cisplatin treatment. To accomplish this, we knocked down CLU in CAL-33 cells and treated these CLU-deficient cells with cisplatin. CLU inhibition reduced cisplatin-induced LC3-II accumulation and successfully rescued SQSTM1 and mitochondrial protein (TOMM20 and COX4I1) turnover compared to shNC cells treated with cisplatin (Figure 2A and S2A_i-v_). Moreover, confocal microscopy-based analysis showed a smaller number of mitochondria colocalized with LC3 puncta per cell in CLU-deficient cells than in shNC-expressing cells treated with cisplatin (Figure 2B). To further confirm the involvement of lysosomes in mitochondrial degradation, we labeled mitochondria and lysosomes using MitoTracker Green and LysoTracker Red, respectively. We found a significant decrease in colocalization of mitochondria with lysosomes in CLU KD cells than in shNC cells in both treated and untreated conditions (Figure 2C), suggesting that endogenous CLU is required for cisplatin-induced mitophagy.

To establish the functional role of CLU in regulating cisplatin-driven mitophagy, we examined mitophagy activity in FaDu-CLU cells in the presence of cisplatin. Notably, FaDu-CLU cells showed rapid conversion of LC3-I to LC3-II and higher reduction of SQSTM1, TOMM20, and COX4I1 levels as compared to FaDu-MOCK cells at the basal level, which was further enhanced in response to cisplatin treatment (Figure 2D and S2B_i-v_). Moreover, confocal microscopy-based colocalization analysis also showed that CLU overexpression led to a higher accumulation of LC3 puncta per cell colocalized with TOMM20 than seen in FaDu-MOCK cells in both cisplatin-treated and untreated conditions (Figure 2E). Even though we could not see the complete colocalization between LC3 and TOMM20 staining, the degree of overlap was identical to that seen in previously reported conditions where mitophagy had been identified [27]. Further, we also noticed a substantial increase in colocalization of mitochondria (MTG) with lysosomes (LTR) in FaDu-CLU cells relative to FaDu-MOCK cells, which further increased after cisplatin treatment (Figure 2F).

**Figure 3.**
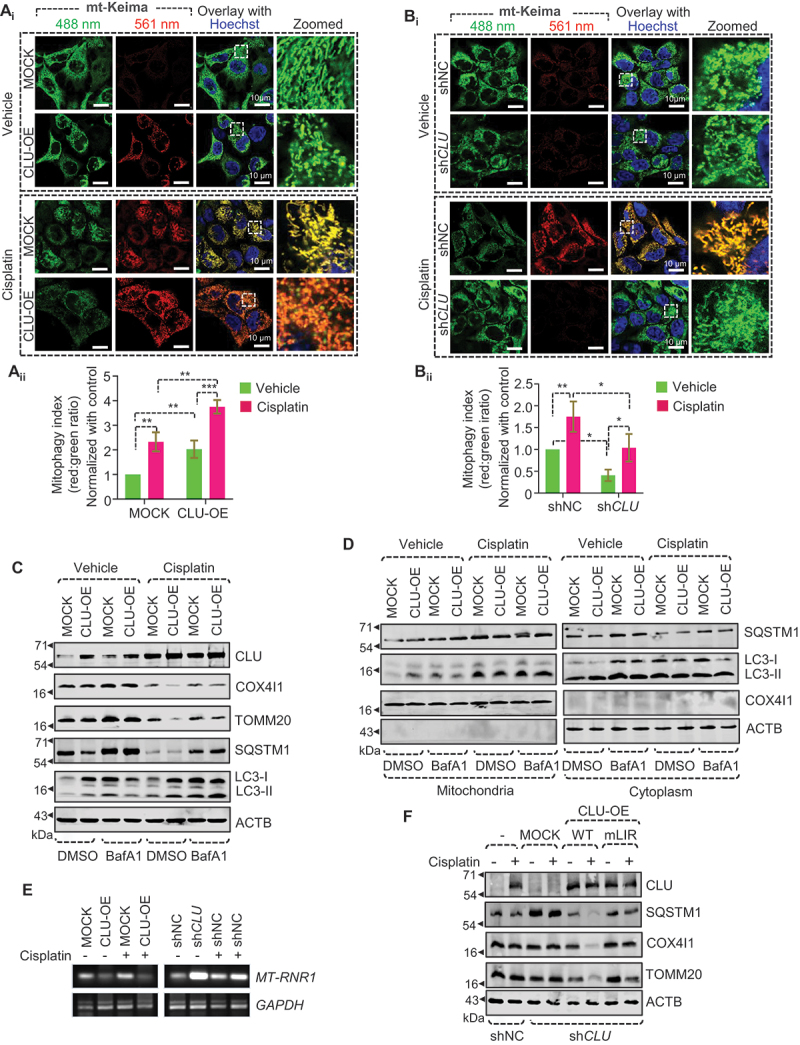
The LIR motif is essential for CLU-mediated clearance of mitochondrial proteins during cisplatin treatment. (A_i_, B_i_) Representative confocal microscopy images of cells with gain and loss of function of CLU stably expressing mKeima-red-Mito7, followed by cisplatin treatment (10 µM; 24 h). (A_ii_, B_ii_) ImageJ-based quantification showing the mitophagy index (red:green ratio) of respective conditions. All the images used for quantification were from three independent experiments. Scale bars: 10 µm. (C) Western blotting analysis showing the mitophagic flux in CLU-OE cells treated with cisplatin combined with BafA1 (mitophagic flux inhibitor). (D) Western blotting analysis of the subcellular fraction isolated from CLU-OE cells treated with cisplatin combined with BafA1. (E) Representative gel image showing the *MT-RNR1* level in CLU-OE or sh*CLU* cells treated with cisplatin (10 µM; 24 h). (F) Western blotting analysis showing the impaired mitophagy status in CLU-deficient cells expressing MOCK, wild-type, and mLIR variant of CLU followed by cisplatin treatment (10 µM; 24 h). Data were normalized to the MOCK cells treated with DMSO (0.1%) (mean ± S.D., *n* = 3). *p < 0.05, **p < 0.01, and ***p < 0.001.

Next, we performed confocal microscopy-based mitophagy flux analysis using mKeima-Red-Mito7, a mitochondria-specific lysosomal hydrolase-resistant probe, which shows a color change from green to red when exposed to acidic pH with a concomitant change in the emission spectrum [28]. Interestingly, a significantly higher mitophagy index (red:green ratio) was observed in FaDu-CLU than in FaDu-MOCK cells, which further increased during cisplatin treatment (Figure 3A). However, CLU KD drastically reduced the mitophagy index suggesting impaired mitophagic flux (Figure 3B). To further confirm CLU-driven mitophagic flux, we applied bafilomycin A_1_ (BafA1, a V-ATPase inhibitor that blocks general autophagy) to prevent the degradation of autolysosome contents in both FaDu-MOCK and FaDu-CLU cells in the presence or absence of cisplatin. Co-treatment of cisplatin+BafA1 resulted in a higher accumulation of LC3-II in FaDu-CLU BafA1 cells than FaDu-MOCK BafA1 cells, and blockage of CLU-mediated degradation of TOMM20 and COX4I1 (Figure 3C and S3A-D) corresponding to inhibition of mitophagic flux. Notably, co-treatment with BafA1 and cisplatin led to a higher accumulation of LC3-II and SQSTM1 in the mitochondrial fraction than cisplatin alone in FaDu-CLU cells (Figure 3D), suggesting that CLU promoted mitophagosome formation, and cisplatin treatment triggered its accumulation to prime mitophagy.

We next examined the effect of gain and loss of function of CLU on mitochondrial DNA (mt-DNA) level and found that CLU overexpression drastically reduced the mt-DNA level, which was triggered during cisplatin treatment. However, CLU knockdown caused a significant increase in the mt-DNA level irrespective of cisplatin treatment (Figure 3E), confirming that CLU is involved in inducing mitophagy in oral cancer cells. The precise recognition and subsequent engulfment of impaired mitochondria necessitate the presence of an operational LIR motif, to facilitate the active interaction between mitophagy receptors and LC3-subfamily proteins [29]. CLU has five LIR-like sequences in the CLU-α-chain, and among the five motifs only _341_YNEL_344_ acts as a putative LIR motif that mediates CLU-LC3 interaction to facilitate autophagy activation [17]. Consequently, we introduced a Y341A L344A double mutant (_341_ANEA_344_; mLIR) and conducted a rescue experiment involving the overexpression of both wild-type and LIR mutants in CLU knockdown cells subjected to cisplatin treatment. We found that the Y341A L344A mLIR successfully reinstated autophagic substrate (SQSTM1) levels alongside mitochondrial proteins (TOMM20 and COX4I1) when compared to wild-type CLU (Figure 3F), signifying the ineffectiveness of the CLU LIR mutant in eliciting cisplatin-induced mitophagy in oral cancer cells. Collectively, these findings underscore the essential role of CLU in the selective elimination of damaged mitochondria through mitophagy in response to cisplatin treatment.

### BAX is required for CLU-mediated clearance of damaged mitochondria in oral cancer cells

**Figure 4.**
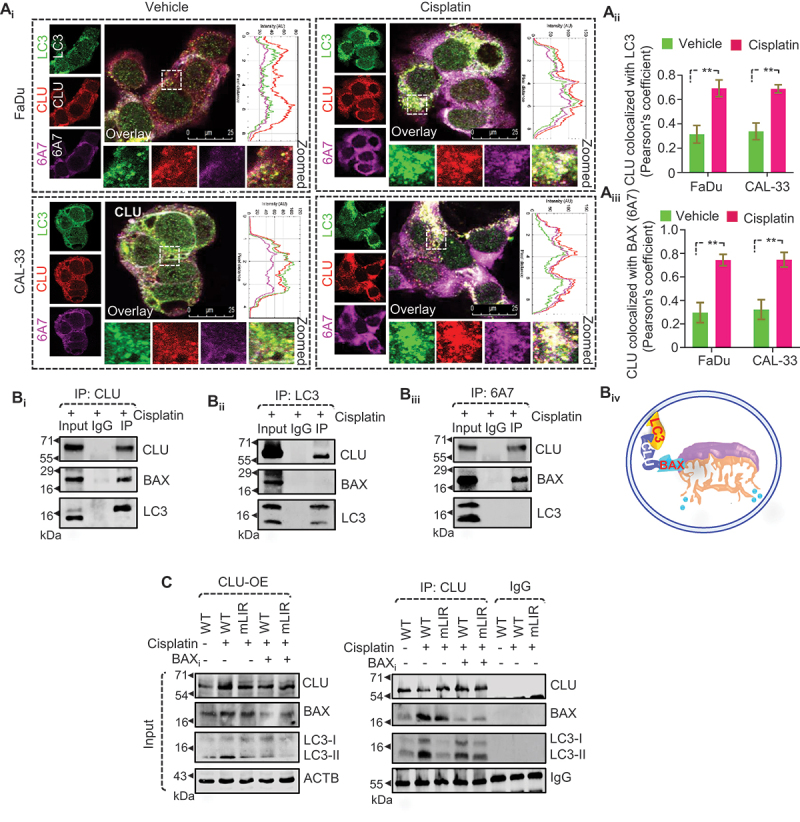
CLU interacts with conformation-altered BAX to remove cisplatin-induced dysfunctional mitochondria. (A_i_) Confocal microscopy-based colocalization analysis of FaDu and CAL-33 cells treated with cisplatin followed by staining with antibodies against LC3 (green), CLU (red), and BAX (6A7) (magenta). Scale bars: 25 µm. (A_ii-iii_) Quantification of colocalization (%) represented as Pearson’s co-efficient value calculated from images of each condition of three independent experiments using the JACoP plugin of ImageJ. (B) Co-immunoprecipitation assay of cisplatin-treated CAL-33 cells using (B_i_) anti-CLU (B_ii_), anti-LC3 and (B_iii_), anti-BAX (6A7) antibodies and detection of CLU, LC3 and BAX in each condition. (B_iv_) Schematic showing the proposed trimeric complex on a damaged mitochondrion within a mitophagosome. (C) Immunoprecipitation assay on FaDu-CLU cells expressing different variants of CLU (wild-type or mLIR) followed by treatment with cisplatin in combination with BAX chelator (50 nm; 3 h) using anti-CLU antibody and detection of CLU, LC3 and BAX in this condition.

CLU plays a crucial role in providing chemoresistance by sequestering BAX to prevent BAX-mediated apoptosis in cancer cells [16]. Moreover, CLU interacts with LC3 to induce autophagosome biogenesis to enhance cancer cell survival [17]. We wondered whether the interaction between CLU-BAX and CLU-LC3 could influence CLU-mediated mitophagy during cisplatin treatment. To explore this possibility, we first performed confocal microscopy-based colocalization analysis using anti-BAX monoclonal antibody clone 6A7, which precisely detects conformation-altered BAX proteins responsible for apoptosis induction [16] along with antibodies against CLU and LC3. Interestingly, cisplatin treatment triggered more substantial colocalization of CLU, LC3, and BAX (6A7) than seen with the untreated groups in FaDu and CAL-33 cells (Figure 4A). Next, to confirm their molecular association, a co-immunoprecipitation assay was performed on cisplatin-treated cells, using combinations of antibodies against the three proteins. Interestingly, we noticed a strong interaction between CLU-BAX, and CLU-LC3. In contrast, there was no interaction between BAX-LC3 (Figure 4B), suggesting that CLU binds both BAX and LC3 but that a trimeric complex is not present at a high level. In fact, the trimeric complex will ultimately be degraded through mitophagy, which may account for our inability to detect it. To further confirm the nature of the molecular interaction (Figure 4B), we performed an immunoprecipitation assay using CLU antibody in cells with different combinations inhibiting BAX activity (using BAX chelator) or CLU-LC3 interaction (through overexpressing the LIR mutant) and compared these with wild-type CLU with/without cisplatin treatment. Intriguingly, we noticed that both inhibitory conditions hindered the CLU-BAX and CLU-LC3 interaction (Figure 4C), establishing that CLU acts as an adaptor protein binding damaged mitochondria via BAX and recruiting the formation of a mitophagosome around it, which presumably requires simultaneous interaction with LC3 (BAX-CLU-LC3) following cisplatin treatment.

Further, we verified the importance of BAX in CLU-mediated clearance of dysfunctional mitochondria through gain- and loss-of-function studies of BAX followed by flow cytometry analysis using MTG and MTR. BAX deficiency through shRNA-mediated inhibition (sh*BAX*) (Figure S4A_i-ii_) triggered a higher accumulation of dysfunctional mitochondria in both FaDu-MOCK and FaDu-CLU cells in response to cisplatin treatment (Figure S4B_i-ii_). However, gain of function of BAX in the DU 145 cell line (BAX negative) (Figure S4C_i-ii_) co-expressing CLU showed a drastic reduction in dysfunctional mitochondria (Figure S4D_i-ii_). As BAX induces mitochondrial outer membrane permeabilization and apoptotic cell death, we further validated our findings in CLU-deficient cells expressing BAX and found that BAX overexpression triggered higher accumulation of dysfunctional mitochondria in the absence of CLU in response to cisplatin treatment (Figure S4E_i-ii_), suggesting there was a higher rate of mitochondrial clearance and a simultaneous increase in mitochondria functionality.

**Figure 5.**
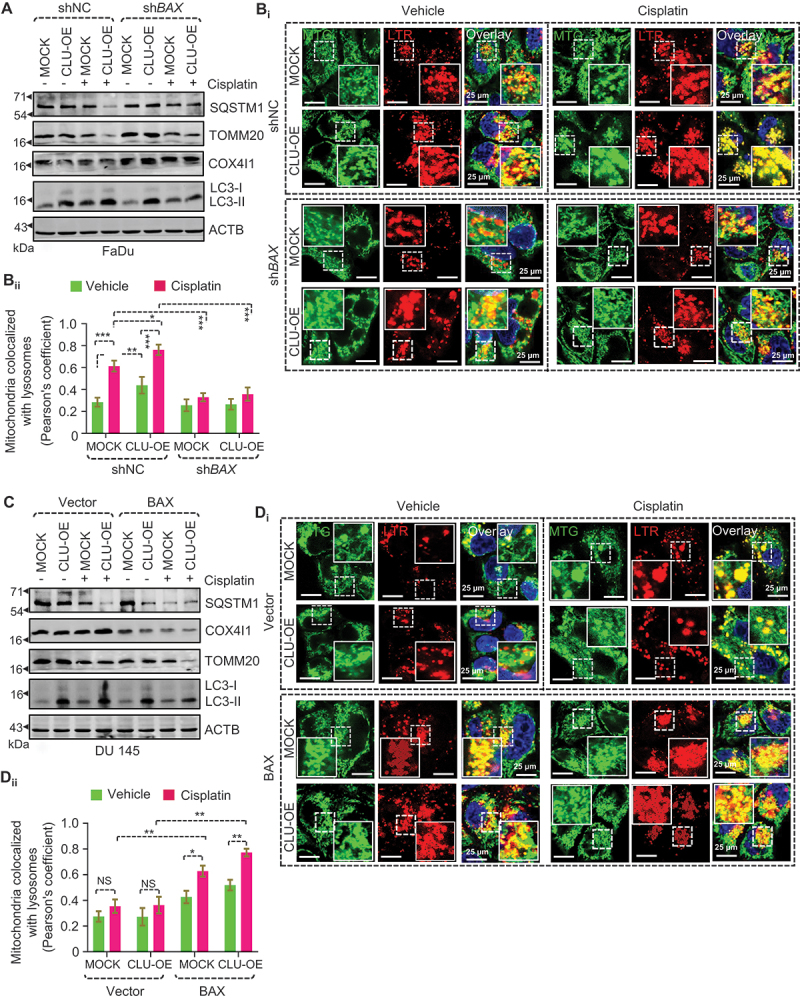
Gain and loss of function of BAX regulate CLU-mediated mitophagy during cisplatin treatment. (A, C) Western blot analysis in CLU-OE FaDu and DU 145 cells with loss of function (transfected with shNC, sh*BAX*) or gain of function (transfected with vector, *BAX*) of BAX followed by cisplatin treatment, respectively. (B_i_, D_i_) Confocal microscopy-based live-cell imaging of cisplatin-treated CLU-OE FaDu and DU 145 cells with loss of function (transfected with shNC, sh*BAX*) and gain of function (transfected with vector, *BAX*) of BAX followed by labelling with LTR (red; representing lysosomes) and MTG (green; representing mitochondria). Scale bars: 25 µm. (B_ii_, D_ii_) Quantification showing colocalization (%) defined as Pearson’s co-efficient value calculated from images of each condition of three independent experiments using the JACoP plugin of ImageJ. Data were normalized to the MOCK or shNC cells (mean ± S.D., *n* = 3). *p < 0.05, **p < 0.01, and ***p < 0.001.

Next, to determine whether CLU-mediated mitophagy was altered in BAX-deficient cells, we treated these BAX-deficient FaDu-CLU and FaDu-MOCK cells with cisplatin and examined the mitophagy status. Interestingly, the inhibition of BAX potently inhibited the elimination of mitochondria in FaDu-CLU cells, as assessed by the detection of TOMM20 and COX4I1 levels, without altering autophagic marker proteins (LC3-II and SQSTM1) (Figure 5A and S5A_i-iv_). Similarly, confocal microscopy-based colocalization analysis also showed that BAX deficiency significantly reduced the colocalization of mitochondria (MTG) with lysosomes (LTR) in both untreated and cisplatin-treated BAX-deficient FaDu-CLU and FaDu-MOCK cells (Figure 5B), indicating impaired mitophagy. In contrast, the gain of function of BAX in DU 145-CLU cell lines resulted in a marked reduction in TOMM20 and COX4I1 levels during cisplatin treatment (Figure 5C and S5B_i-iv_). At the same time, no noticeable change in the mitochondrial turnover could be seen even after cisplatin treatment in both DU 145-MOCK and DU 145-CLU cells expressing empty vector. Moreover, BAX overexpression significantly increased the fusion of mitochondria (MTG) with lysosomes (LTR), as indicated by a higher number of yellow dots with a more elevated Pearson’s coefficient after cisplatin treatment in DU 145-CLU cells than in vector-expressing DU 145-CLU cells (Figure 5D). Altogether, our findings indicate the crucial role of BAX in CLU-mediated mitophagy in the elimination of damaged mitochondria during chemotherapeutic stress.

### Inhibition of mitophagic flux promotes CLU-mediated mitophagosome accumulation to induce ROS-dependent mitochondrial apoptosis

**Figure 6.**
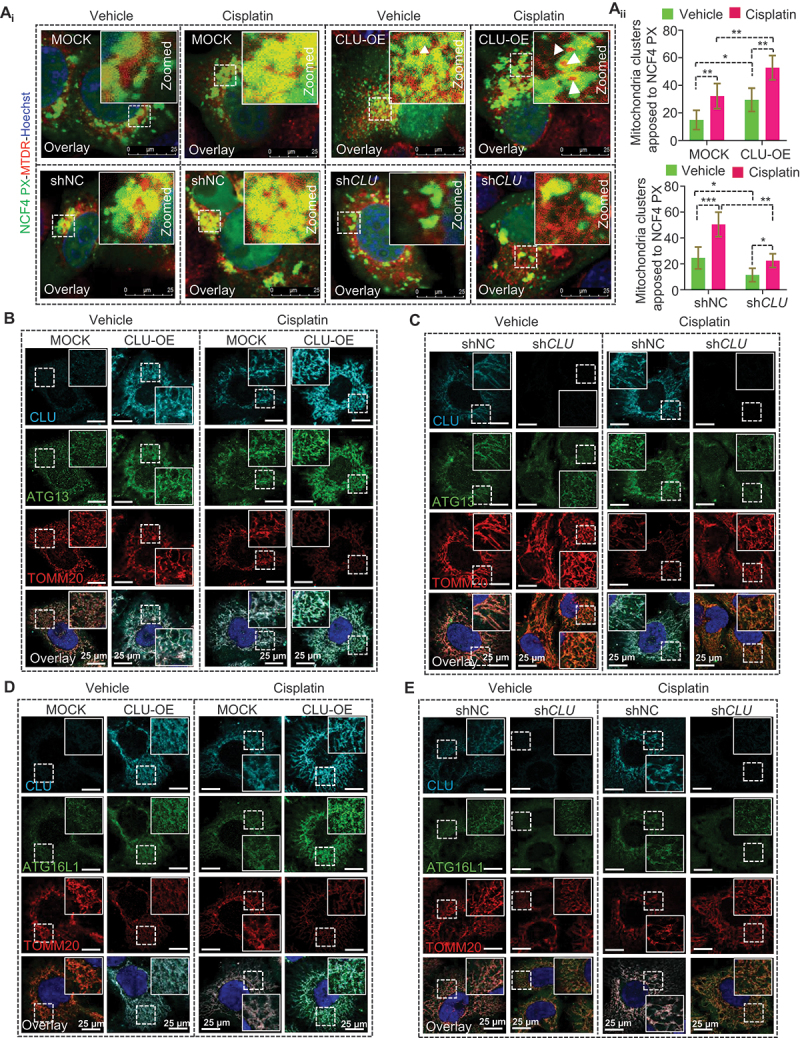
CLU promotes ATG16L1 and ATG13 puncta accumulation near mitochondria during cisplatin treatment in oral cancer cells. (A_i_) Confocal microscopy-based colocalization analysis in CLU-OE and CLU KD cells transiently expressing an NCF4/p40(phox) PX-EGFP plasmid followed by cisplatin (10 µM; 24 h) treatment and labeled with MitoTracker^TM^ Deep Red (MTDR; representing mitochondria). The magnified image represents the boxed area. (A_ii_) Quantification of the number of cells with perinuclear mitochondrial clusters in contact with NCF4 PX-EGFP hotspots denoted as a percentage of the total number of mitochondria-containing cells. All images from three independent experiments were randomly taken for each condition and analyzed through ImageJ software. Scale bars: 25 µm. (B-E) Representative confocal microscopy-based colocalization analysis in CLU-OE and CLU KD cells treated with cisplatin (10 µM; 24 h) followed by staining with (B, C) CLU (cyan), ATG13 (green), and TOMM20 (red), (D, E) CLU (cyan), ATG16L1 (green), and TOMM20 (red). The magnified image represents the boxed area. All images represent three independent experiments and were randomly taken for each condition and analyzed through ImageJ software. Scale bars: 25 µm.

Activating the class III PtdIns3K is an important event for initiating the phagophore membrane to induce autophagy. We hypothesized that CLU recruitment might be responsible for activating class III PtdIns3K adjacent to damaged mitochondria. To test this hypothesis, we ectopically expressed NCF4/p40 (phox) PX-EGFP, which binds specifically to PtdIns3P [30], in both CLU-overexpressing (OE) and CLU KD cells and monitored the subcellular distribution of PtdIns3P in response to cisplatin treatment. Interestingly, CLU overexpression led to more NCF4 PX-EGFP hotspots formed around depolarized mitochondrial clusters during cisplatin treatment. Along these lines, CLU knockdown substantially reduced the total number of NCF4 PX-EGFP hotspots per cell without altering mitochondrial clustering (Figure 6A). Moreover, we also noticed that gain and loss of function of CLU also altered the ATG13 and ATG16L1 puncta level and their distribution around mitochondria as observed by lower Pearson coefficient values than MOCK or shNC cells in response to cisplatin treatment (Figure 6B-E, S6A-D). This finding indicates that CLU triggers class III PtdIns3K activation to initiate phagophore membrane formation around damaged mitochondria during chemotherapeutic stress.

**Figure 7.**
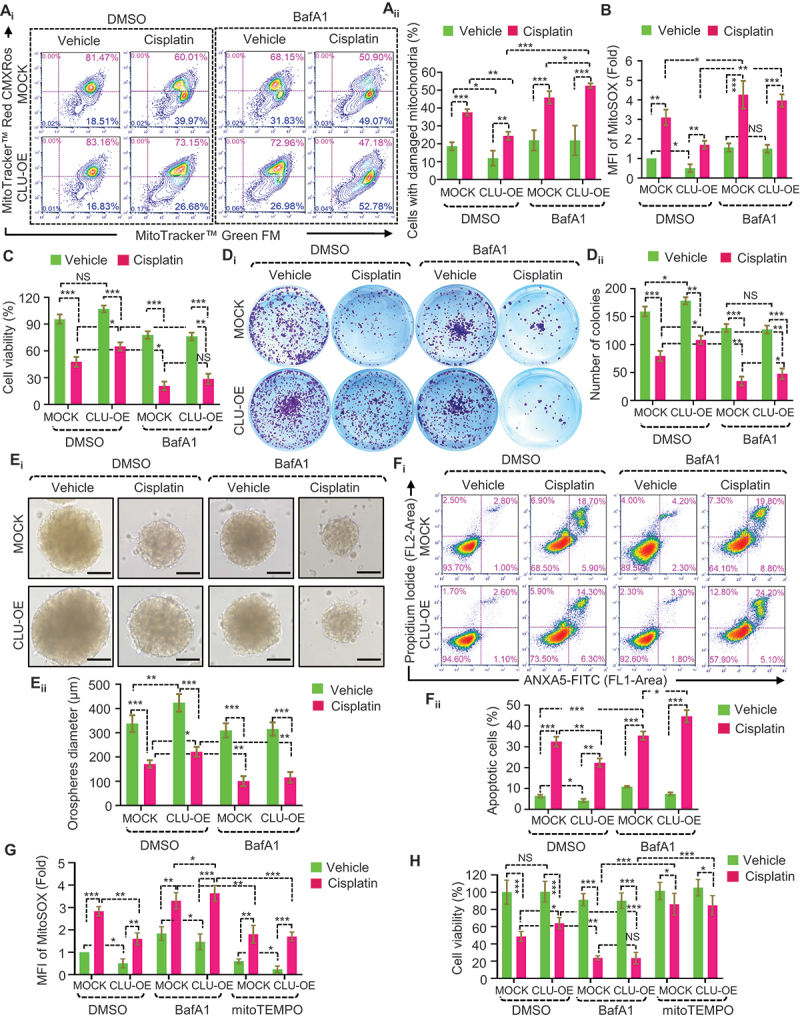
Inhibition of mitophagy flux triggers mtROS-dependent apoptosis in oral cancer cells. (A-F) FaDu-MOCK or FaDu-CLU cells treated with cisplatin (10 µM; 24 h) in combination with BafA1 (50 nM; 3 h). (A_i-ii_) Flow cytometry analysis to assess mitochondrial dysfunction using MitoTracker Green (MTG) and MitoTracker Red CMXRos (MTR) (mean ± S.D., *n* = 3) followed by its quantification. (B) Flow cytometry analysis to quantify the mitochondrial superoxide levels, as indicated by MitoSOX fluorescence (mean ± S.D., *n* = 3). MFI, mean fluorescence intensity. (C) Cell viability using MTT assay (mean ± S.D., *n* = 5). (D_i-ii_) Clone-forming ability, and its quantification (mean ± S.D., *n* = 3). (E_i_) Representative images of orospheres formed after MOCK or CLU-OE cells were treated with vehicle control, cisplatin and BafA1, followed by culturing for 7 days. Scale bar: 200 μm. (E_ii_) Quantifications of orosphere diameter numbers formed after treatment (mean ± S.D., *n* = 25). **p < 0.01, and ***p < 0.001. (F_i-ii_) Flow cytometry analysis to access apoptosis using ANXA5-PI staining, followed by its quantification (mean ± S.D., *n* = 3). (G) Flow cytometry analysis to quantify the mitochondrial superoxide levels, as indicated by MitoSOX fluorescence in MitoTEMPO (mitochondrial superoxide mimetic)-pretreated FaDu-MOCK or FaDu-CLU cells treated with cisplatin (10 µM; 24 h) in combination with BafA1 (50 nM; 3 h), (mean ± S.D., *n* = 3). (H) Cell viability assay estimating the growth rate in FaDu-MOCK or FaDu-CLU cells treated with cisplatin+BafA1 in the presence or absence of mitoTEMPO. Data were normalized to the untreated cells of each group (mean ± S.D., *n* = 5). *p < 0.05, **p < 0.01, and ***p < 0.001.

We extended our analysis using flow cytometry with MTG and MTR and found that inhibition of mitophagic flux impaired the clearance of damaged mitochondria and triggered a higher accumulation of dysfunctional mitochondria in both FaDu-MOCK and FaDu-CLU cells in response to cisplatin treatment (Figure 7A). As accumulated damaged mitochondria trigger reactive oxygen species (ROS) production, we measured ROS levels in the indicated treatment conditions. As expected, cisplatin treatment triggered ROS production. More importantly, co-treatment with BafA1+cisplatin markedly increased ROS levels in both FaDu-MOCK and FaDu-CLU cells (Figure 7B and S7A-B). Next, we examined the effect of excessive accumulation of mitophagosomes on cancer cell growth and survival. Co-treatment with BafA1+cisplatin significantly increased cytotoxicity, as indicated by a substantial reduction in cell viability (Figure 7C) and the number of clones (Figure 7D) in both FaDu-MOCK and FaDu-CLU cells. Moreover, ANXA5-PI staining confirmed the presence of more apoptotic cells during combined treatment (cisplatin+BafA1) than cisplatin alone in FaDu-CLU cells (Figure 7E). In addition, BafA1 treatment also increased the expression of apoptosis markers as indicated by the lower expression of anti-apoptotic marker BIRC2, and higher expression of cleaved PARP (Figure S7C_i-iv_). Similarly, co-treatment of BafA1+cisplatin also showed enhanced CASP3-CASP7 activity in both FaDu-CLU and FaDu-MOCK cells (Figure S7D), confirming the activation of cisplatin-induced apoptosis. Next, we chose a three-dimensional (3D) tumor spheroid assay to better mimic in vivo tumorigenesis and evaluated the synergistic effect of co-treatment on cell growth. As expected, FaDu-CLU cells showed bigger size orospheres than FaDu-MOCK cells in both treated and untreated groups; however, co-treatment (cisplatin+BafA1) substantially decreased orosphere diameter in both FaDu-CLU and FaDu-MOCK cells (Figure 7F). More importantly, treatment with mitoTEMPO, (a mitochondria-targeted SOD [superoxide dismutase] mimetic) and N-acetyl-L-cysteine (NAC, a general ROS scavenger), significantly reduced the ROS production (Figure 7G, S7E) and its associated cytotoxic effect (Figure 7H, S7F) in the co-treatment (BafA1+cisplatin) groups. Collectively, our results suggest that inhibition of CLU-mediated mitophagy flux could function as a novel mechanism to potentiate cisplatin treatment in oral cancer cells.

### PPARGC1A maintains mitochondrial biogenesis during CLU-induced mitophagy in oral cancer cells

**Figure 8.**
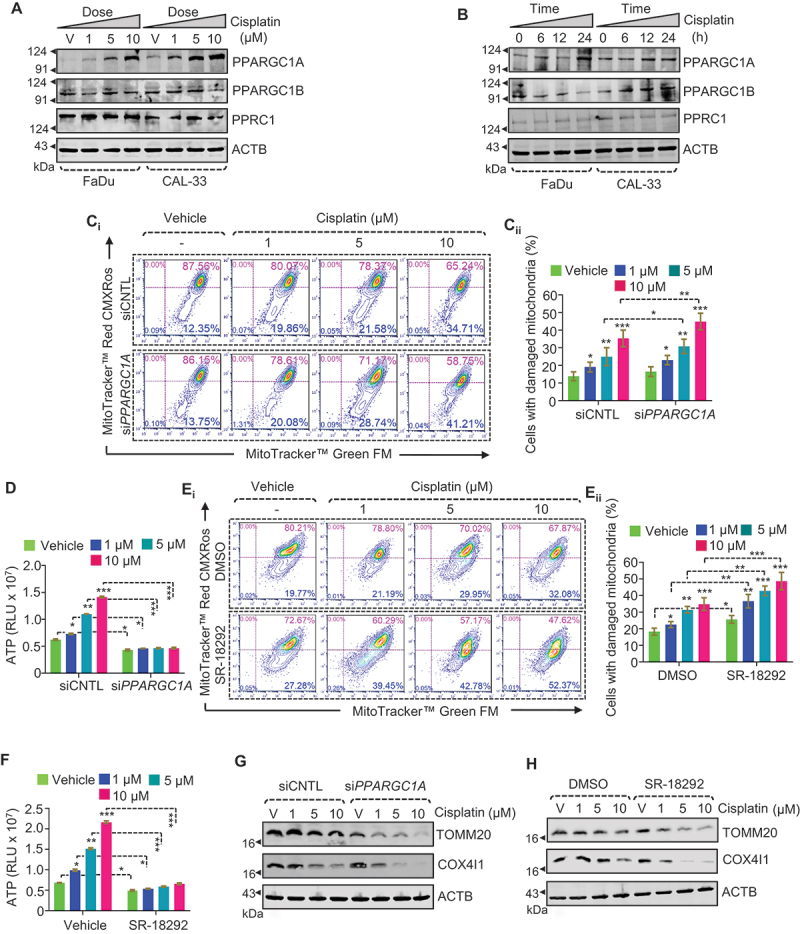
Cisplatin triggers PPARGC1A-mediated mitochondrial biogenesis to maintain mitochondrial content. (A-B) Western blotting analysis showing the expression of PPARGC1A, PPARGC1B, and PPRC1 in both FaDu and CAL-33 cells treated with (A) various concentrations of cisplatin (1, 5, and 10 μM; 24 h), (B) for different time-points (0, 6, 12, and 24 h). (C_i_, E_i_) Flow cytometry analysis to assess mitochondrial dysfunction using MitoTracker Green (MTG) and MitoTracker Red CMXRos (MTR) in PPARGC1A-deficient (si*PPARGC1A* and SR-18292 treated) and control (siCNTL and DMSO treated) cells treated with different concentrations of cisplatin (1, 5, and 10 μM; 24 h). (C_ii_, E_ii_) Quantification showing the cells with damaged mitochondria (%) (mean ± S.D., *n* = 3). (D, F) ATP measurement in PPARGC1A-deficient (si*PPARGC1A* and SR-18292 treated) and control (siCNTL and DMSO treated) cells treated with different concentrations of cisplatin (1, 5, and 10 μM; 24 h). (G, H) Western blotting analysis showing the expression of TOMM20, and COX4I1 in PPARGC1A-deficient (si*PPARGC1A* and SR-18292 treated) and control (siCNTL and DMSO treated) cells treated with different concentrations of cisplatin (1, 5, and 10 μM; 24 h) (mean ± S.D., *n* = 3).

Mitochondrial biogenesis could be activated to compensate for the mitochondrial loss that occurs following exposure to cisplatin. Accordingly, we examined the former during cisplatin treatment. To accomplish this, we monitored the transcriptional coactivator PPARGC1A, a chief regulator of mitochondrial biogenesis [8], along with other PPARGC family members such as PPARGC1B/PGC1β and PPRC1/PRC. Of note, cisplatin treatment substantially increased the expression of PPARGC1A in both dose- and time-dependent manners (Figure 8A-B, S8A_i-iii_, and S8B_i-iii_). In contrast, other PPARGC family members, such as PPARGC1B and PPRC1, remained relatively unchanged. Next, we knocked down PPARGC1A using small interfering RNA (si*PPARGC1A*) and downregulation of expression was confirmed through western blot analysis (Figure S8C). Genetic inhibition of PPARGC1A significantly increased damaged mitochondria during cisplatin treatment (1, 5, 10 µM; 24 h) (Figure 8C), which caused a sharp fall in mitochondrial ATP levels (Figure 8D). Similarly, co-treatment with different concentrations of cisplatin (1, 5, 10 µM)+SR-18292 (a pharmacological inhibitor of PPARGC1A) [31] also led to a higher number of damaged mitochondria than seen with DMSO-treated cells (Figure 8E), along with a drastic fall in the mitochondrial ATP level (Figure 8F). More importantly, PPARGC1A-deficient (si*PPARGC1A* or SR-18292 treated) cells showed a more rapid mitochondrial turnover and, in particular with si*PPARGC1A,* than control cells treated with cisplatin (1, 5, 10 µM; 24 h) (Figure 8G-H, S8D_i-ii,_ S8E_i-ii_). In addition, we also noticed that PPARGC1A-deficient (si*PPARGC1A* or SR-18292 treatment) cells significantly increased cisplatin-induced cytotoxicity, as indicated by lower cell viability than control (siCNTL or DMSO treated) cells during cisplatin treatment (Figure S8F-G). This finding suggests that PPARGC1A-induced mitochondrial biogenesis maintains mitochondrial homeostasis for oral cancer cell survival.

### PPARGC1A inhibition alters mitochondrial biogenesis triggering excessive loss of mitochondrial mass in CLU-overexpressing oral cancer cells

To establish the correlation between CLU and PPARGC1A in regulating mitochondrial functionality, we measured the alteration in their expression in oral cancer cells. Notably, loss of function of CLU did not alter the expression of PPARGC1A during cisplatin treatment (Figure S9A_i-ii_). Similarly, PPARGC1A knockdown did not change endogenous CLU levels during cisplatin treatment (Figure S9B_i-ii_). Moreover, gain and loss of function of CLU could not alter cisplatin-driven ATP generation (Figure S9C-D), confirming that cisplatin independently activates PPARGC1A-mediated mitochondrial biogenesis irrespective of the presence of CLU.

**Figure 9.**
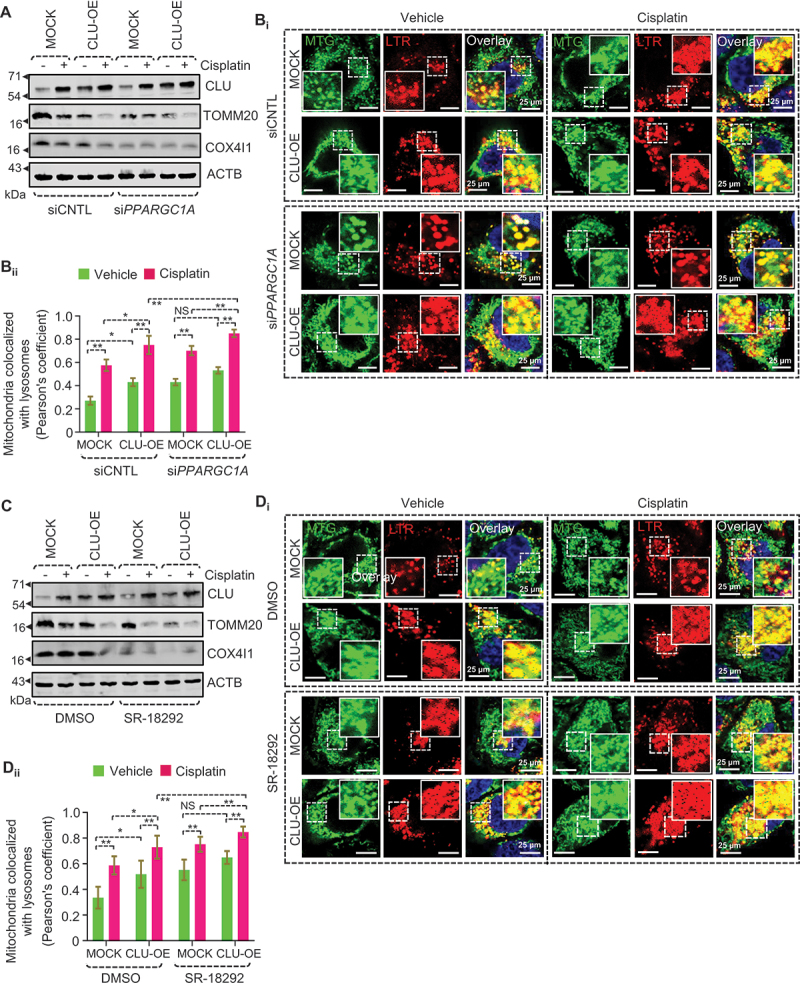
Inhibition of PPARGC1A caused excessive loss of mitochondrial mass through exaggerating CLU-mediated mitophagy. PPARGC1A-deficient (si*PPARGC1A* and SR-18292 treated) and control (siCNTL and DMSO treated) MOCK or CLU-OE cells were treated with cisplatin (10 μM; 24 h), followed by (A, C) western blotting analysis showing the expression of TOMM20, and COX4I1. (B_i_, D_i_) Confocal microscopy-based colocalization analysis for LysoTracker Red (LTR; representing lysosomes) and MitoTracker Green (MTG; representing mitochondria). (B_ii_, D_ii_) Quantification represents the colocalization of MTG with LTR from the confocal microscopy-based live-cell image analysis using Pearson’s co-efficient value calculated from images of each condition taken from three independent experiments using the JACoP plugin of ImageJ. Scale bar: 25 μm. Error bars: S.D. *p < 0.05, **p < 0.01.

Next, we wondered how PPARGC1A inhibition affects mitophagy status in CLU-overexpressing cells in response to cisplatin treatment. Interestingly, PPARGC1A knockdown further enhanced mitochondrial turnover in cisplatin-treated FaDu-CLU cells as indicated by a marked reduction in TOMM20 and COX4I1 (Figure 9A, S9E_i-ii_). Moreover, PPARGC1A inhibition promoted efficient fusion of lysosomes (LTR) with mitochondria (MTR) as indicated by stronger colocalization (Pearson’s co-efficient) in CLU-OE cells than in MOCK cells (Figure 9B). To extend this analysis, we treated CLU-OE cells with SR-18292 and found that SR-18292 treatment further enhanced mitochondrial turnover in CLU-OE cells than MOCK cells during cisplatin treatment (Figure 9C and S9F_i-ii_). Moreover, co-treatment of SR-18292+cisplatin also triggered the fusion of mitochondria with lysosomes, as indicated by MTG-LTR colocalization analysis in cisplatin-treated CLU-OE cells (Figure 9D). Next, we examined OCR, and mitochondrial ATP levels and found that PPARGC1A knockdown also reduced the OCR level (Figure S9G) and mitochondrial ATP (Figure S9H) in CLU-OE cells during cisplatin treatment. Moreover, similar to genetic inhibition, SR-18292 treatment triggered OCR inhibition (Figure S9I) and reduced mitochondrial ATP (Figure S9J) in cisplatin-treated FaDu-CLU cells compared to FaDu-MOCK cells. Cumulatively, our results suggest that CLU and PPARGC1A act independently, regulating mitophagy and mitochondrial biogenesis to fine tune mitochondrial content for cell survival.

### PPARGC1A inhibition increases susceptibility to cisplatin through mitophagy-associated cell death in CLU-overexpressing oral cancer cells

**Figure 10.**
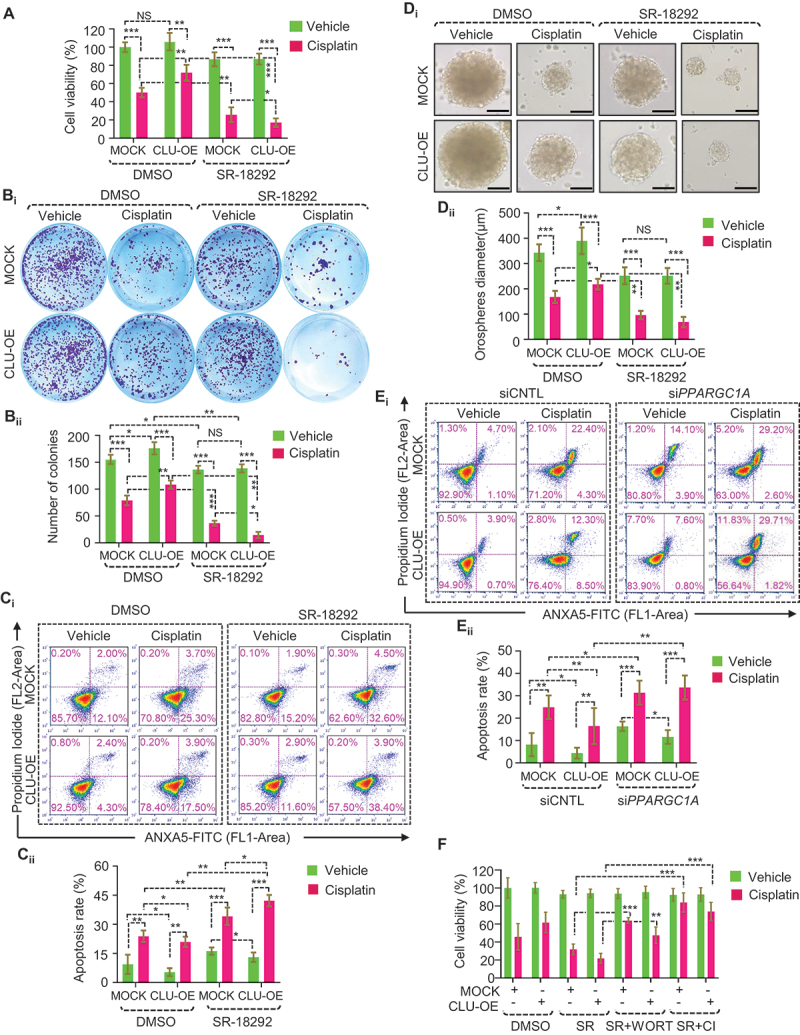
SR-18292 synergistically improves cisplatin-mediated cytotoxicity in oral cancer cells. (A-E) PPARGC1A-deficient and control (siCNTL and DMSO treated) MOCK or CLU-OE cells were treated with cisplatin (10 μM; 24 h), followed by (A) cell viability measurement using an MTT assay to estimate the growth rate, and (B_i-ii_) clone-forming ability and its quantification (mean ± S.D., *n* = 3). (C_i_, E_i_) Flow cytometry analysis to access apoptosis using ANXA5-PI staining, followed by its quantification (mean ± S.D., *n* = 3), and (C_ii_, E_ii_) quantification showing the apoptosis rate in respective conditions (mean ± S.D., *n* = 3). (D_i-ii_) Representative images of orospheres formed after MOCK or CLU-OE cells were treated with vehicle control, or cisplatin alone or in combination with SR-18292, followed by culturing for 7 days. Scale bar: 200 μm. (D_ii_) Quantifications of orospheres diameters formed after treatment (mean ± S.D., *n* = 25). *p < 0.05, **p < 0.01, and ***p < 0.001. (F) Cell viability assay to monitor PPARGC1A-deficient and control (DMSO treated) MOCK or CLU-OE cells treated with cisplatin (10 μM; 24 h), in combination with wortmannin (50 nM) or caspase inhibitor (CI; 50 nM). Data were normalized to the untreated cells of each group (mean ± S.D., *n* = 5). **p < 0.01, and ***p < 0.001.

As cisplatin promotes PPARGC1A-mediated mitochondrial biogenesis, we next sought to examine whether abrogation of mitochondrial biogenesis reversed CLU-dependent mitophagy-mediated cytoprotection *in vitro*. To accomplish this, we measured cancer growth, survival, and apoptosis using different apoptotic markers in PPARGC1A-deficient (si*PPARGC1A* and SR-18292 treatment) FaDu-MOCK or FaDu-CLU cells during cisplatin treatment. The co-treatment of SR-18292+cisplatin in both FaDu-MOCK and FaDu-CLU cell lines significantly reduced the cell viability, although a limited cytotoxic effect could also be seen in the cisplatin (alone) or SR-18292 groups (Figure 10A). In addition, we noticed that co-treatment of SR-18292+cisplatin also reduced clonogenic survivability more than cisplatin alone in both FaDu-MOCK and FaDu-CLU cells (Figure 10B).

Next, we explored apoptosis involvement through ANXA5-PI staining and CASP3-CASP7 assay. Notably, the ANXA5-PI staining assay confirmed that more apoptotic cells could be seen in the co-treatment group than with cisplatin alone in FaDu-MOCK and FaDu-CLU cells (Figure 10C). Similarly, this synergistic inhibitory effect of SR-18292+cisplatin was also reflected in a 3D tumor spheroid formation assay (Figure 10D). A similar observation of a significantly higher cisplatin-induced cell death (ANXA5^+^ PI^−^ and ANXA5^+^ PI^+^) was noticed in PPARGC1A-deficient FaDu-CLU or FaDu-MOCK cells than the remarkably resistant FaDu-CLU cells during cisplatin treatment (Figure 10E). Of note, PPARGC1A-deficient (si*PPARGC1A* and SR-18292 treatment) cells showed higher CASP3-CASP7 activity in both FaDu-MOCK and FaDu-CLU cells, irrespective of CLU status (Figure S10A-B). Moreover, to delineate the types of cell death activated during co-treatment with SR-18292+cisplatin, we treated both MOCK or CLU-OE cells with wortmannin (50 nM; 3 h), which inhibits PtdIns3K and prevents autophagy initiation and Z-VAD-FMK (50 nM; 3 h), a pan-caspase inhibitor (CI). Interestingly, co-treatment with wortmannin significantly reduced the cytotoxic effect of SR-18292+cisplatin, as indicated by higher cell viability. Similarly, pretreatment with CI also improved cell viability in both MOCK or CLU-OE cells (Figure 10F), suggesting that co-treatment with SR-18292+cisplatin triggered mitophagy-associated cell death in oral cancer cells. These data collectively highlight the potential benefit of combining PPARGC1A inhibitor (SR-18292) with current chemotherapy drugs such as cisplatin, shifting the balance toward excessive mitophagy-associated cell death in oral cancer cells *in vitro*.

### PPARGC1A inhibitor SR-18292 and cisplatin synergistically inhibit oral cancer cell growth in a mouse xenograft model

**Figure 11.**
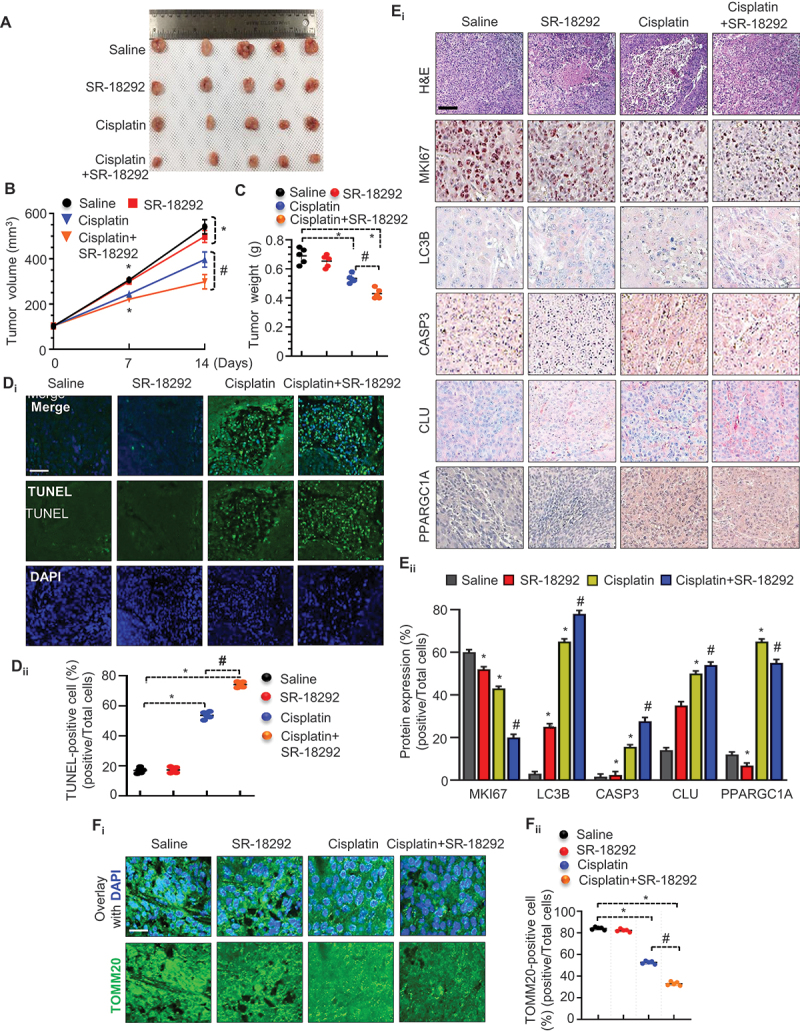
Cisplatin activates mitophagy-associated cell death in the absence of mitochondrial biogenesis *in vivo*. (A) Tumor volume was monitored every other day during treatments: vehicle (DMSO), SR-18292 (20 mg/kg), cisplatin (1 mg/kg), or SR-18292 plus cisplatin for 14 days. At the end of the treatment period, the tumors were taken out and photographed. (B) The graph shows changes in the tumor volume. The data represented are the means ± S.D. (*n* = 5). (C) The size and weight of the dissected tumors are shown. (D_i-ii_) Representative images of tumor sections that were analyzed by TUNEL assay. DAPI is used to stain nuclei. Scale bar: 50 μm. TUNEL-positive cells were quantified by counting nuclei in five randomly chosen fields. (E_i_) The tumor sections were subjected to H&E staining and immunohistochemistry staining for MKI67, LC3, CASP3, CLU, and PPARGC1A, scale bar: 100 μm. (E_ii_) Quantification shows the expression of the indicated proteins. (F_i-ii_) Representative microscopy images of tumor sections immunostained with TOMM20. DAPI is used to stain nuclei. Scale bar: 50 μm. All images were taken from a randomly chosen field. *p < 0.05, #p < 0.01.

To establish the *in vivo* efficacy of targeting mitochondrial biogenesis through SR-18292 in oral cancer cells, we developed a subcutaneous tumor model in immunocompromised mice using the FaDu cells. We noticed a substantial reduction in tumor growth and size after combined treatment (SR-18292+cisplatin) compared with the single-treatment groups (SR-18292or cisplatin) (Figure 11A), which is consistent with our *in vitro* findings in oral cancer cells. More importantly, body weight remained relatively the same among mice of different treatment groups. Of note, SR-18292 or cisplatin treatment alone showed a negligible effect on the growth of the tumor. However, co-treatment with SR-18292 and cisplatin showed a synergistic effect and significantly reduced the tumor size compared to both control groups and cisplatin- or SR-18292-treated (alone) groups (Figure 11B-C). Morphological changes were also monitored in the excised tumor samples through hematoxylin and eosin (H&E) staining, which showed significant differences in morphology, with signs of necrosis in co-treatment groups.

Further, to delineate the cause of cell death in tumors, we performed a TUNEL assay in both control and treated groups. Cisplatin or SR-18292 treatment alone increased the number of TUNEL-positive cells (green color). More importantly, the co-treatment (cisplatin+SR-18292) significantly increased the TUNEL-positive cell count compared to either control or treatment-alone groups (Figure 11D). In addition, the co-treatment with SR-18292 and cisplatin also exaggerated both autophagy-associated cell death as indicated by stronger immunoreactivity for CASP3 (apoptosis marker) and LC3 (autophagy marker) (Figure 11E). Moreover, we also noticed that co-treatment groups drastically reduced TOMM20 expression (Figure 11F). Collectively, these data indicated that pharmacological inhibition of mitochondrial biogenesis (SR-18292) synergistically increased cisplatin cytotoxicity, inhibiting tumor growth of oral cancer *in vivo* and could be useful for optimal clinical efficacy.

## Discussion

Cancer cells preserve accurate mitochondrial activity by promoting mitochondrial turnover through mitophagy and generating a renewable pool of new and healthy mitochondria through mitochondrial biogenesis [32–34]. Studies have reported that mitophagy could be either protective or detrimental depending on the context. For instance, excessive or sustained mitophagy is associated with increased cell death [25,35]. The molecular mechanisms behind the coordinated regulation of these opposing processes and the detrimental effect of excessive mitophagy without proper biogenesis in cancer cells are not well understood.

During stress, mitophagy receptors including BNIP3, BNIP3L, and FUNDC1 sense the stress signal and are activated to remove dysfunctional mitochondria, thus maintaining quality control and cellular homeostasis. Here, we found that CLU translocation to mitochondria protects against cisplatin-induced mitochondrial damage, consistent with previous findings. In this connection, we found that mitochondrial redistribution of CLU maintains mitochondrial membrane integrity, which is essential for the anti-apoptotic functions of CLU [36]. Moreover, like other well-known mitophagy receptors such as BNIP3L and FUNDC1 [37,38], CLU overexpression triggers mitophagy to clear damaged mitochondria, protecting cancer cells from cisplatin-induced stress. More importantly, chemotherapeutic stress promotes the interaction between CLU and conformation-altered BAX to inhibit apoptosis [16]. Similarly, a previous report showed that CLU triggers autophagosome biogenesis through its interaction with LC3, promoting LC3 lipidation through a stable LC3-ATG3 hetero-complex to acquire anticancer drug resistance [17]. Intriguingly, we noticed that CLU interacts with both LC3 and BAX, presumably forming a trimeric complex (BAX-CLU-LC3) in cisplatin-treated cells recruiting a phagophore to generate a mitophagosome around damaged mitochondria for successful mitophagy leading to enhanced oral cancer cell survival. Excessive accumulation of mitophagosomes contributes to mitochondrial injury and apoptosis [39]. Our study showed that CLU promotes such excessive accumulation of mitophagosomes during impaired mitophagy flux, causing mitochondrial ROS-dependent apoptosis in oral cancer.

Mitochondrial biogenesis is mainly regulated by key transcription factors such as PPARGC1A, PPARGC1B, and PPRC1, which then co-activate several downstream transcription factors, including ESSRA/ERRα, NRF1, and NFE2L2 [40]. Interestingly, we observed that cisplatin triggers PPARGC1A activity, whereas other transcription factors such as PPRC1 and PPARGC1B remain unchanged. PPARGC1A positively regulates cancer growth metastasis, but inhibition significantly reduces mitochondrial number, cell proliferation, and survival against ROS-driven apoptosis in cancer cells [23,41]. For example, enhanced expression of PPARGC1A triggers mitochondrial oxidative metabolism providing chemoresistance in melanoma cells [22]. Our results confirm the idea that PPARGC1A inhibition enhanced cisplatin-induced mitochondrial dysfunction, which triggers mitochondrial (mt)ROS-dependent apoptosis in oral cancer cells. Several research findings support the conclusion that PPARGC1A protects against ROS-driven apoptosis through activating antioxidant enzymes [42,43]. Similarly, our results also showed that PPARGC1A inhibition leads to a drastic decrease in mitochondrial protein and downstream transcription cofactors of mitochondrial biogenesis such as NRF1 and NFE2L2, indicating PPARGC1A is a critical regulator, stimulating cisplatin-driven mitochondrial biogenesis.

Mitochondrial biogenesis and mitophagy are critical opposing processes essential for mitochondrial homeostasis. A previous study showed the dichotomous role of PPARGC1A, where it enhances mitochondrial mass through mitochondrial biogenesis and buffers the ROS- and FOXO1-mediated expression of genes to regulate mitophagy [44]. Interestingly, PPARGC1A, along with NRF1, activates FUNDC1 to induce mitophagy to maintain quality control of mitochondria [45]. In another study, IGF1 (insulin like growth factor 1) signaling activates mitochondrial biogenesis and mitophagy via BNIP3 expression to sustain cancer cell growth and viability [46]. Our study showed that CLU and PPARGC1A coordinately regulate mitophagy and biogenesis to enhance cell survival in oral cancer during cisplatin treatment. More importantly, inhibition of PPARGC1A in CLU-overexpressing cells disturbs the mitochondrial homeostasis resulting in excessive mitophagy and mitophagy-associated cell death during exposure to cisplatin. Our results confirmed that co-treatment with PPARGC1A inhibitor and cisplatin potently increased cisplatin-induced cell death and exhibited enhanced antitumor activity in FaDu xenograft models. Similar to our results, a previous report showed that cisplatin treatment triggered apoptosis in ovarian cancer [47]. Comprehensively, with these findings, we propose that simultaneous targeting of both CLU-mediated mitophagy and PPARGC1A-dependent mitochondrial biogenesis could be helpful in overcoming drug resistance to cisplatin and enhancing mitophagy-associated cell death for better therapeutic outcomes in oral cancer.

## Conclusion

**Figure 12.**
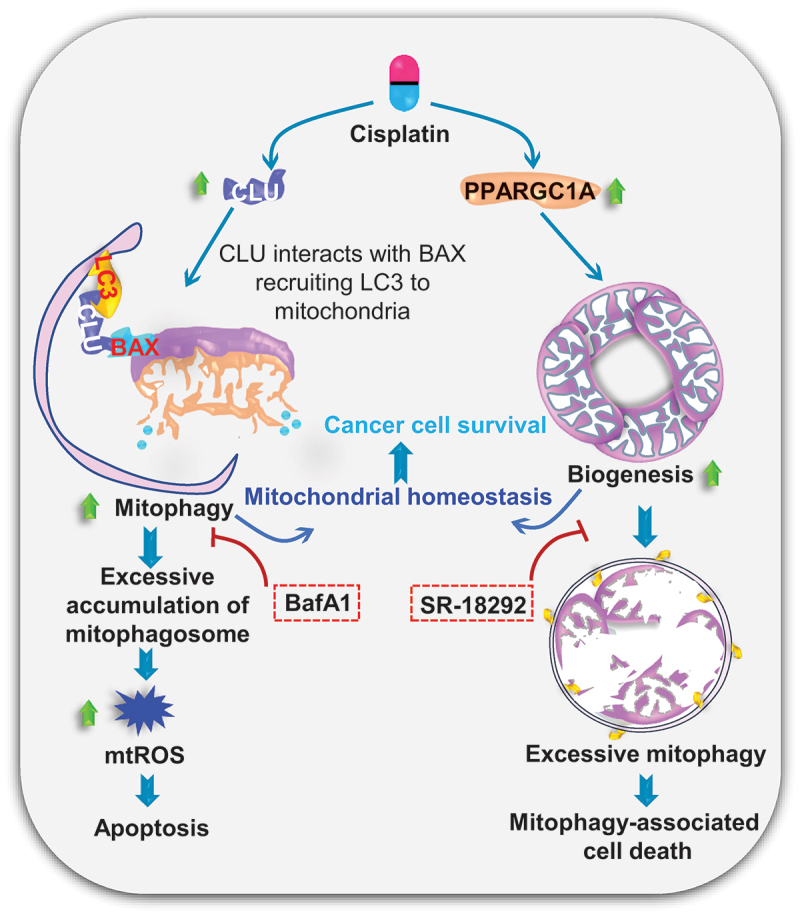
Proposed mechanism by which CLU and PPARGC1A coordinately regulate mitochondrial homeostasis through activating mitophagy and mitochondrial biogenesis, respectively. CLU interacts with activated BAX and LC3 recruiting a mitophagosome around damaged mitochondria to enhance mitophagic flux during chemotherapeutic stress. Moreover, chemotherapeutic stress also activates PPARGC1A-mediated mitochondrial biogenesis in oral cancer cells. Inhibition of CLU-mediated mitophagy leads to the accumulation of damaged mitochondria, generating excessive mitochondrial superoxide leading to apoptosis. Similarly, targeted inhibition of PPARGC1A-mediated mitochondrial biogenesis triggers excessive mitophagy, sensitizing oral cancer cells toward mitophagy-associated cell death.

In conclusion, we have revealed that CLU-mediated mitophagy is coupled with PPARGC1A-dependent mitochondrial biogenesis to fine tune mitochondrial homeostasis. Defective mitochondrial biogenesis leads to excessive mitochondrial turnover activating mitophagy-associated cell death in oral cancer (Figure 12). We suggest that CLU and PPARGC1A are indispensable for controlling mitochondrial content during chemotherapeutic stress and that modulating their activity could strongly influence therapeutic responses in oral cancer.

## Materials and methods

### Reagents and antibodies

Bafilomycin A_1_ (BafA1; Calbiochem, 196000), cisplatin (cis-diammineplatinum [II] dichloride; Sigma-Aldrich, P4394), DCFDA (Sigma-Aldrich, D6883), mitoTEMPO (Sigma-Aldrich, SML0737), N-acetyl-L-cysteine (NAC; Sigma-Aldrich, A9165), SR-18292 (Sigma-Aldrich, SML2146) and wortmannin (Tokyo Chemical Industry, W0007) are commercially available.

The ACTB/β-Actin (BD Biosciences, 612656; 1:10000), BAX (6A7) (Santa Cruz Biotechnology, sc -23959; 1:200 for IP and 1:250 for IF), BAX (Santa Cruz Biotechnology, sc-20067; 1:500), BIRC2/cIAP1 (Cell Signaling Technology, 7065S; 1:1000), CASP3 (Cell Signaling Technology, 9662S; 1:1000), CLU/clusterin (Santa Cruz Biotechnology, sc-166907; 1:1000), COX4I1/COXIV (Cell Signaling Technology, 4844S; 1:6000), MKI67/Ki67 (Santa Cruz Biotechnology, sc-23900; 1:250), LC3 (Cell Signaling Technology, 83506S; 1:1000 for WB and 1:500 for IF), LC3 (Sigma-Aldrich, L7543; 1:500 for IF), LC3 (Novus Biologicals, NB100–2220; 1:300 for IHC), SQSTM1/p62 (Cell Signaling Technology, 88588S; 1:1000), PARP (Cell Signaling Technology, 9542S; 1:1000), PPARGC1A/PGC1α (Santa Cruz Biotechnology, sc-517380; 1:500 for WB), PPARGC1A/PGC1α (Santa Cruz Biotechnology, sc-518025; 1:200 for IHC), PPARGC1B/PGC1β (Santa Cruz Biotechnology, sc -517279; 1:500), PPRC1/PRC (Santa Cruz Biotechnology, sc-376431; 1:500), TOMM20 (BD Biosciences, 612278; 1:6000 for WB and 1:3000 for IF), TOMM20 (Abcam, ab186735; 1:250 for IF), anti-mouse IgG Alexa Fluor 568 (Thermo Fisher Scientific, A-11004; 1:500), anti-rabbit IgG Alexa Fluor 568 (Thermo Fisher Scientific, A-11011; 1:500), anti-rabbit IgG Alexa Fluor 488 (Thermo Fisher Scientific, A-11008; 1:500), anti-mouse IgG Alexa Fluor 488 (Thermo Fisher Scientific, A-11001; 1:500), anti-mouse IgG Alexa Fluor® 647 (Abcam, ab150115; 1:500), and anti-rabbit IgG Alexa Fluor 700 (Thermo Fisher Scientific, A-21038; 1:500) antibodies are commercially available.

### Cell lines culture

FaDu (ATCC; HTB-43, RRID: CVCL_1218) and DU 145 (ATCC; HTB-81, RRID: CVCL_0105) were obtained from the American Type Culture Collection. CAL-33 (DSMZ, ACC-447, RRID: CVCL_1108) was generously provided by Dr Goutam Sethi (National University of Singapore). These cell lines were cultured in their respective media (FaDu: Minimum Essential Medium [MEM; Himedia, AL047]; CAL-33: Dulbecco’s Modified Eagle’s Medium [DMEM; Himedia, AL151A]; DU 145: Roswell Park Memorial Institute [RPMI 1640; Himedia, AL162S]) medium using standard cell culture techniques. FBS (10%; Gibco 10270106) and antibiotic-antimycotic solution (Himedia, A002A) were added to each media before culturing the cells.

### Generation of stable cell lines and shRNA- or siRNA-mediated knockdown studies

FaDu cells (50,000 cells/well) were grown in 24-well plates using standard cell culture techniques to generate stable cell lines. Cells were transfected (60–70% confluent) with the indicated constructs corresponding to the empty vector (MOCK) or encoding CLU using Lipofectamine 3000 (Invitrogen, L3000001) following the recommended instructions. After transfection, cells were selected using hygromycin B (250 µg/ml; Sigma-Aldrich, H3274), and selected cells were further expanded in the presence of hygromycin B for all experimental studies [19,48].

For knockdown studies, cells were transfected with small-hairpin RNA specifically targeting human *CLU* (sh*CLU*) (Sigma-Aldrich, SHCLND), sh*BAX* (Addgene, 16575; deposited by Bert Vogelstein), and shNC (non-targeting shRNA) from (Sigma-Aldrich, SHC016) and siRNAs such as si*BECN1* (Santa Cruz Biotechnology, sc-29797), si*PPARGC1A/PGC1α* (Santa Cruz Biotechnology, sc-38884) and scrambled siRNA (siCNTL) (Santa Cruz Biotechnology, sc-37007), using Lipofectamine® 3000 reagent (ThermoFisher Scientific, L3000015) following the manufacturer’s instruction. The pCEP4-HA-BAX plasmid (Addgene, 16587; deposited by Bert Vogelstein) was used for gain of function of BAX and mKeima-Red-Mito-7 plasmid (Addgene, 56018; deposited by Michael Davidson) was used for mitophagic flux analysis.

### Western blot analysis

After treatment, cells were harvested and mixed with cell lysis buffer (Cell Signaling Technology, 9803), supplemented with phosphatase (Sigma-Aldrich, P0044) and protease (Sigma-Aldrich, P8340) inhibitors and ruptured through vigorous mixing for 2 h on ice. Then the whole content was subjected to centrifugation (12000 × g) for 10 min and the supernatant was collected. The Bio-Rad Protein Assay kit (Bio-Rad, 5000006) was used to quantify the protein concentration in the collected supernatant. The whole-cell lysates (35 μg of protein) were mixed with 4× Laemmli buffer (Bio-Rad, 1610747) supplemented with β-mercaptoethanol (0.1%; HIMEDIA, RM2895; v:v) and boiled for 5 min. SDS-PAGE was used to separate the proteins, and then transferred onto nitrocellulose membranes (PALL Life Sciences, 66485) at 100 V for 2 h. Then nitrocellulose membrane was incubated with nonfat milk (5%; HIMEDIA, M530; w:v) for membrane blocking (1 h; room temperature [RT]) with continuous shaking with subsequent probing at 4°C with the respective primary antibodies. Following overnight incubation, the membranes were washed with PBST (PBS [HIMEDIA, TL1006] + Tween 20^TM^ [Sigma- Aldrich, P9416; 0.03%]); 3 × 10 min) followed by probing with HRP-conjugated secondary antibodies (Abgenex Pvt. Ltd., 1:5000; 2 h in the dark; RT). Finally, the protein bands were detected using ECL detection reagent (Bio-Rad, 1705062).

### Subcellular fractionation

Cells were lysed using a Cell Fractionation Kit (Abcam, ab109719), following the instructions given in the kit. In brief, cells were first trypsinized and pelleted by centrifugation at 300 × g for 5 min. Then cells were resuspended in an equal volume of Buffer A and Buffer B and incubated for 7 min at RT with constant mixing, followed by sequential centrifugation at 5000 × g and 10,000 × g for 1 min each at 4°C to obtain the cytosolic fraction. For the mitochondrial fraction, the cytosolic pellet was again resuspended in Buffer C to an equal volume as Buffer A, followed by constant mixing for 10 min at RT. The pellets were then sequentially centrifuged at 5000 × g and 10,000 × g for 1 min each at 4°C to obtain the mitochondrial fraction. The respective fractions were then used for western blot analysis with GAPDH and COX4I1 as a cytosolic and as a mitochondrial purity marker, respectively.

### Confocal microscopy-based colocalization analysis

For colocalization studies, cells after drug exposure were incubated (15 min; RT) with formaldehyde (Sigma-Aldrich, 252549) (4% v:v; 15 min at RT), followed by blocking and permeabilization with bovine serum albumin (BSA; 5% w:v; HIMEDIA, MB083) + Triton X-100 (0.03% v:v; Sigma-Aldrich, X100) for 1 h at RT. Cells were then incubated with the respective primary antibody prepared in BSA (1%, w:v) + Triton X-100 (0.03%, v:v) overnight at 4°C. The next day, the tagged cells were rinsed three times with PBS and followed by incubation with conjugated secondary antibodies (6 h; RT) in the dark. Last, cells were counterstained with DAPI for 5 min followed by PBS rinsing (5 × 5 min) and immediately imaged. For live-cell colocalization studies, cells were marked with MitoTracker Green (50 nM; Invitrogen, M7514) and LysoTracker Red (50 nM; Invitrogen, L7528), followed by a 30-min incubation (37°C; 5% CO_2_) and counterstained with Hoechst 33342 solution (1 µg/ml; Sigma-Aldrich, B2261) for 5 min. Then cells were rinsed with PBS (5 × 5 min) and immediately imaged. All the images were captured using a Leica confocal laser scanning microscope followed by image analysis using ImageJ software and the appropriate tools. For colocalization analysis, the JACoP plugin of ImageJ software was used, and colocalization was expressed as Pearson’s overlap coefficient. The sample size is mentioned as *n* value for each experiment. All images were randomly taken for each sample using a 63X objective lens of a Leica confocal microscope (Leica Microsystems; Wetzlar, Germany), as previously described [49,50].

### Co-immunoprecipitation assay

For the co-immunoprecipitation assay, 350 μg of protein were immune-precipitated using a Dynabeads™ Protein A Immunoprecipitation Kit (Invitrogen™, 10006D). In brief, the beads were conjugated with respective primary antibodies for 30 min with constant agitation at RT, followed by washing with wash buffer supplied with the kit. The beads with individual primary antibodies were then resuspended with 350 μg of protein overnight at 4°C. The next day, the formed immune complexes were gently washed three times, followed by elution with elution buffer (20 µl) supplied with the kit. Next, the eluted samples were mixed with 4× Laemmli sample buffer, boiled for 10 min at 70°C for denaturation, and subjected to western blot analysis. Normal IgG (Santa Cruz Biotechnology, sc-2025) was combined with 350 µg of protein in the respective treatment conditions for negative controls.

### Site-directed mutagenesis for generating the CLU-LIR mutant

The QuikChange II XL site-directed Mutagenesis Kit (Agilent Technologies, 200521) was used to generate CLU mutants according to the manufacturer’s instruction. Primers used to generate the mutants were obtained from IDT (Integrated DNA Technologies, CA, USA): Forward: 5”-GCTGAGAGGTTGACCAGGAAAGCCAACGAGGCGCTAAAGTCCTACCAG-3‘; reverse: 5’-CTGGTAGGACTTTAGCGCCTCGTTGGCTTTCCTGGTCAACCTCTCAGC-3”. The presence of the desired mutations was confirmed by DNA sequencing before proceeding to further experimental analysis.

### Mitochondrial DNA isolation and quantification

For mitochondrial DNA quantification, total DNA containing genomic DNA and mitochondrial DNA was purified from cells. Mitochondrial DNA was then quantified by PCR using primers that targets the mitochondrial *MT-RNR1/12S* rRNA. The level of mitochondrial DNA was then normalized against nuclear *GAPDH* DNA, which served as the internal control for comparative analysis [51].

### Estimation of mitochondrial health

For estimating mitochondrial health, transfected cells (50,000/well) were cultured in 24-well plates using standard cell culture techniques. Then cells were exposed to respective drugs (cisplatin [10 µM], SR-18292 [10 µM], bafilomycin A_1_ [50 nM; 3 h]) alone or in combination for 24 h. Following incubation, cells were then labeled with two mitochondrial-specific dyes MitoTracker Green (100 nM) and MitoTracker Red CMXRos (Invitrogen, M7512; 100 nM) and incubated for 30 min at 37°C in the dark. After completion, cells were trypsinized and resuspended in PBS solution (1% FBS), and mitochondrial health status was verified using a flow cytometer (BD Accurri C6 Software; RRID: SCR_001456). Forward scatter versus side scatter plots were used to eliminate debris, while FL1-Area (MitoTracker Green) and FL2-Area (MitoTracker Red) were used for further analysis. Each experiment was carried out at least three times, and a representative result was shown. All the data were analyzed through FCS Express™ software 7 (De Novo Software, Glendale, CA, USA).

### Measurement of mitochondrial respiration

OCR was measured using an Oxygen Consumption Rate Assay Kit (Cayman, 600800), according to the manufacturer’s instruction. In brief, cells (30,000 cells/well) were seeded in a black, clear-bottom 96-well plate (Thermo Scientific™, 165305) in 100 μl of culture medium for 14 h before the start of the experiment, followed by the addition of 150 μl fresh media with the desired concentration of cisplatin for the indicated conditions. Next, MitoXpress Xtra Solution (10 μl) was added to each well, followed by adding 100 μl of warm mineral oil gently over the top of each well to prevent the supply of oxygen. Then, the change in fluorescence signals was measured at excitation (380 nm)/emission (650 nm) using a fluorescence plate reader (Synergy H1 Hybrid Microplate Reader, Biotek).

### ATP measurement

For measuring cellular ATP content, the ENLITEN® ATP Assay System Bioluminescence kit (Promega, FF2000) was used following the manufacturer’s instruction. In brief, after treatments, cells were harvested and lysed, followed by protein quantification. Equal concentration of proteins (whole-cell lysate) was mixed with an equal volume of the reagents supplied in the kit and subjected to the luminescence measurement using a GloMax® 20/20 Luminometer (Promega Corp, Fitchburg, WI, USA). The mean of three independent experiments was used for statistical analysis, and the estimated values were represented as a mean of relative light units (RLU).

### Estimation of intracellular and mitochondrial ROS generation

Cells were harvested after drug treatments and resuspended with 100 μl of PBS containing 2′,7′-dichlorofluorescin diacetate (10 µM; Sigma-Aldrich, D6283), followed by incubation (37°C, 30 min) in the dark for monitoring intracellular ROS generation. Similarly, DHE (1 μM; Sigma-Aldrich, 37291) and MitoSOX Red (5 μM; Invitrogen, M36008) were used for monitoring extra-mitochondrial and intra-mitochondrial superoxide production, respectively. After adding the respective fluorescent dye, cells were incubated for 30 min at 37°C in the dark, followed by washing (PBS) and data acquisition. The amount of both intracellular and mitochondrial ROS generation was determined using a flow cytometer and FCS Express™ software 7, as previously described [49].

### Monitoring apoptosis rate through flow cytometry analysis

FaDu-MOCK and FaDu-CLU cells were exposed to respective drugs (cisplatin [10 µM], SR-18292 [10 µM], bafilomycin A_1_ [50 nM; 3 h]) alone or in combination for 24 h, and control cells (untreated) were exposed to DMSO (0.01% v:v). After completion, cells were trypsinized, washed (ice-cold PBS), stained with dual ANXA5 (ImmunoTools, 31490013X2) and propidium iodide (PI) (Sigma-Aldrich, P4170) staining and subjected to flow cytometry analysis (BD Accuri C6™ flow cytometer). Forward scatter versus side scatter plots were used to eliminate debris, while FL1-Area (ANXA5-FITC) and FL2-Area (PI) were used for further analysis. All the data were analyzed through FCS Express™ software 7, as previously described [49].

### Measurement of CASP3-CASP7 activity

For measuring caspase activity, the Caspase-Glo® 3/7 Assay kit (Promega Corp, G8090) was used according to the manufacturer’s protocol. In brief, after cell lysis 25 μg of protein (whole-cell lysate) was mixed with the supplied standard reaction buffer containing the peptide substrate (1:1; v:v). The mixture was then incubated (2 h; RT in the dark) with constant shaking. After completion, the mixed content was subjected to the luminescence measurement using a GloMax® 20/20 Luminometer. The mean of three independent experiments was used for statistical analysis, and the estimated values for CASP3-CASP7 activity were represented as a mean of RLU.

### Cell viability assay

For the cell viability assay, approximately 3000 cells/well of both FaDu and CAL-33 cells were cultured in 96-well plates, cultured overnight, and subjected to respective treatments for the desired times. After specific time points, MTT (3-[4,5-dimethylthiazol-2-yl]-2,5-diphenyltetrazolium bromide; 5 mg/ml, 20 µl; Sigma-Aldrich, M2128) was added to each well and further incubated in a CO_2_ incubator for an additional 2 h. After incubation, the media was carefully decanted from each well, followed by adding DMSO (200 µl; Sigma-Aldrich, D8418) to solubilize formazan crystals and further incubated for an additional 20 min in the dark at RT followed by an absorbance reading at 595 nm. The drug sensitivity (alone or in combination) was calculated as a percentage of cell viability relative to untreated controls, which was considered to be 100%.

### Clonogenic survival assay

For monitoring clonogenicity, FaDu-CLU, FaDu-MOCK, CAL-33-sh*CLU*, and CAL-33-shNC cells (25,000/well) were cultured in 24-well plates using standard cell culture techniques. Following overnight incubation, stably transfected cells were exposed to 50 nM bafilomycin A_1_ for 3 h, 10 µM SR-18292, and 10 µM cisplatin for 24 h (alone or in combination). Then, cells were harvested, and approximately 1000 cells/well were again seeded in another 6-well plate for the next 10 days. The cells were then exposed to paraformaldehyde (4%) for fixation (30 min, at RT), followed by crystal violet (HIMEDIA, GRM961; 0.1%) staining (30 min, at RT) and rinsing with tap water. The stained colonies were imaged and counted manually. The mean of three independent experiments was used for statistical analysis.

### 3D sphere-formation assay

For 3D sphere formation, FaDu cells stably expressing CLU and MOCK were first cultured in 24-well plates (25,000 cells/well) in their optimal culture conditions, followed by subsequent drug treatment. After treatment, cells were trypsinized, and about 2000 cells/well were grown in stem-cell media (serum-free media + B27 [1:50; Gibco, 17504044] + N2 supplement [1%; Gibco, 17502048] + human FGF2 [10 ng/ml; Gibco, PHG0024] + human EGF [20 ng/ml; Gibco, PHG0311]) formulated as previously described [50] in ultra-low attachment 6-well plates (Corning®, CLS3473) for 7 days. After treatment, the 3D spheroid was imaged through an Olympus IX71 fluorescence inverted microscope with a 20X objective (Bright-field mode), and their size (diameter) was measured through cell Sens imaging software. The mean of three independent experiments was used for statistical analysis.

### Animal studies

Six- to eight-week-old BklNbt:BALB/c/nu/nu mice (Damul, Daejeon, Korea) were used for tumor xenografts. Mice were housed (*n* = 5/cage) in a fully climate-controlled room at constant temperature and humidity on a 12:12 h light/dark cycle with free access to food and water. Animal experiments were carried out in compliance with the guidelines set by the Jeonbuk National University hospital animal care and use committee (cuh-IACUC-2019-22). FaDu cells growing exponentially in culture were trypsinized and quantified by trypan blue exclusion, and 3–5 × 10^6^ cells were resuspended in 0.1 ml PBS. The cells were subcutaneously injected into the flank of each mouse. When the tumor reached a weight of ~100 mg (7‒10 days after inoculation), mice were randomly assigned to receive SR-18292, cisplatin, or SR-18292 with cisplatin. After 18 days, mice were euthanized, solid tumors were dissected, and tumor volume was recorded. Tumor size was measured with calipers. Tumor volume (mm^3^) was calculated with the formula: (shortest diameter)^2^ × (longest diameter)/2. Mice were evaluated twice weekly and were sacrificed by cervical dislocation when they showed signs of terminal illness such as hind leg paralysis and inability to eat or drink, and/or were moribund.

### Histological analysis and immunohistochemistry (IHC) analysis

Tissue was dissected and fixed in 10% formalin. Fixed paraffin-embedded samples were cut at 4-µm thickness and stained with hematoxylin and eosin (H&E). For IHC, tissues were fixed in 10% neutral-buffered formalin and embedded in paraffin, cut into 4-μm-thick sections, and immunofluorescence staining of tissues was performed as previously reported [52]. Briefly, sections were incubated with DAKO peroxidase blocking solution (Agilent Technologies, S202386–2) for 10 min at room temperature and washed with TBS-T buffer (25 mM Tris, pH 7.4, 3.0 mM KCl, 140 mM NaCl and 0.05% Tween™ 20) followed by protein blocking. The sections were incubated overnight at 4°C with antibodies against MKI67/Ki-67, LC3B, CASP3, CLU, and PPARGC1A/PGC1α diluted in Dako Antibody Diluent (Agilent Technologies, S3022). The sections were subsequently incubated with Envision + System-HRP labeled Polymer anti-mouse (Agilent Technologies, K4001) secondary antibody. Staining color was developed by Dako AEC High Sensitivity Substrate Chromogen (Agilent Technologies, K3461).

### Immunofluorescence

Paraffin-embedded 4-μm-thick sections were deparaffinized, rehydrated, and incubated in 1% BSA for 2 h at room temperature. The sections were incubated with antibody against TOMM20 at 4°C overnight. Later, the sections were labeled using anti-rabbit IgG-FITC (Sigma Aldrich, F9887). Finally, the nuclei were counterstained with DAPI, samples were mounted, and images captured using the EVOS M5000 Cell Imaging System (Thermo Fisher Scientific, MA, USA).

### TUNEL assay

The terminal deoxynucleotidyl transferase UTP nick-end labeling (TUNEL) assay was performed using the in-situ Cell Death Detection Kit (Roche, 12156792910). In brief, tumor sections were deparaffinized and rehydrated in a series of ethanol (100%, 95%, and 70%) until distilled water is used. Next, sections were incubated with proteinase K solution (10 μg/mL; Biosesang, PF2029-105-00) for 20 min at RT, followed by rinsing with PBS (2 times). Then, the TUNEL reaction mixture (Enzyme solution and Label solution, ratio 1:9) was added to the sections and incubated for 60 min at 37°C in humidified conditions in the dark, followed by rinsing with PBS (3×5 min). The slides were then mounted with DAPI and observed using confocal laser scanning microscopy. Cells were counted in 3–5 high-power fields of each tissue section (*n* = 6) and the percentage TUNEL-positive cells (green) was calculated.

### Statistical analysis

GraphPad Prism-9 (GraphPad Prism, RRID: SCR_002798) was used for statistical analyses. The error bars in the figures denote the mean ±S.D. (standard deviation) of three independent experiments. Differences between groups were measured using a two-tailed paired t-test when the number of groups was two and one-way ANOVA (followed by Bonferroni posttest analysis) where the number of groups was more than 2. *p < 0.05, **p < 0.01, and ***p < 0.001 were considered statistically significant for all experiments. ImageJ software was used for densitometric analysis of western blotting bands.

## Abbreviations

ACTB: actin beta
BafA1: bafilomycin A_1_
BIRC: baculoviral IAP repeat containing
CLU: clusterin
COX4I1: cytochrome c oxidase subunit 4I1
CQ: chloroquine
CYCS: cytochrome c, somatic
DMSO: dimethyl sulfoxide
KD: knockdown
LIR: LC3-interacting region
LTR: LysoTracker Red
mt-DNA: mitochondrial DNA
MTG: MitoTracker Green
MTDR: MitoTrackerTM Deep Red
NAC: N-acetyl-L-cysteine
OCR: oxygen consumption rate
OE: overexpressing
PPARG/PPARγ: peroxisome proliferator activated receptor gamma
TOMM20: translocase of outer mitochondrial membrane 20.

## Supplementary Material

Revised_Supplementary_Files_Praharaj_et_al_2023_R2.docx
