# Supplementary material for: CLU (clusterin) and PPARGC1A/PGC1α coordinately control mitophagy and mitochondrial biogenesis for oral cancer cell survival
Source: Autophagy. 2024 Mar 6;20(6):1359–82. doi: 10.1080/15548627.2024.2309904 (PMC11210931; doi:10.1080/15548627.2024.2309904)
Supplement: Revised_Supplementary_Files_Praharaj_et_al_2023_R2.docx [file KAUP_A_2309904_SM7829.docx]

**
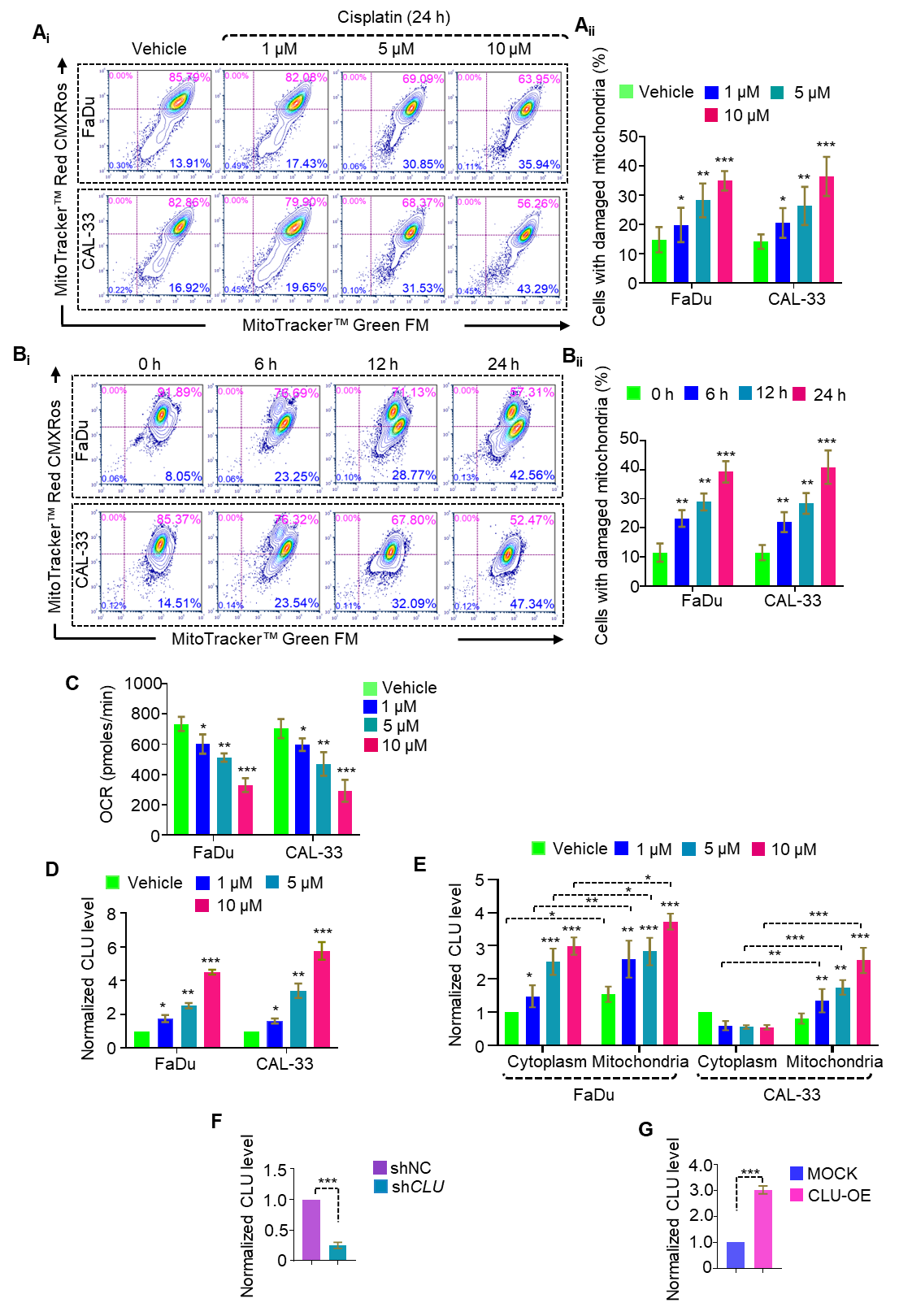
**

**Figure S1.** Cisplatin triggered mitochondrial dysfunction and CLU expression in oral cancer cells. (**A_i,_ B_i_**) FaDu and CAL-33 cells were treated with different concentrations of cisplatin (0, 1, 5, and 10 µM) for 24 h or 10 µM cisplatin for different time points (0, 6, 12, and 24 h), followed by flow cytometry analysis using MitoTracker Green (MTG) and MitoTracker Red CMXRos (MTR) to access damaged mitochondrial status. (**A_ii,_ B_ii_**) Quantification showing cells with damaged mitochondria (%). (**C**) OCR analysis on FaDu and CAL-33 cells treated with different concentration of cisplatin for 24 h. (**D, E**) Densitometric analysis showing the expression of CLU in whole-cell lysates and after subcellular fractionation of cisplatin-treated (1, 5, and 10 µM) FaDu and CAL-33 cells. (**F, G**) Western blot quantification in FaDu and CAL-33 cells with loss of function (transfected with shNC, sh*CLU*) and gain of function (stably expressing MOCK, CLU-OE). ACTB was used as a loading control and for fold change calculation. Data were normalized to the shNC or MOCK untreated cells or the vehicle group (mean ± S.D., n = 3). Error bars: S.D. ∗p < 0.05, ∗∗p < 0.01, ∗∗∗p < 0.001.


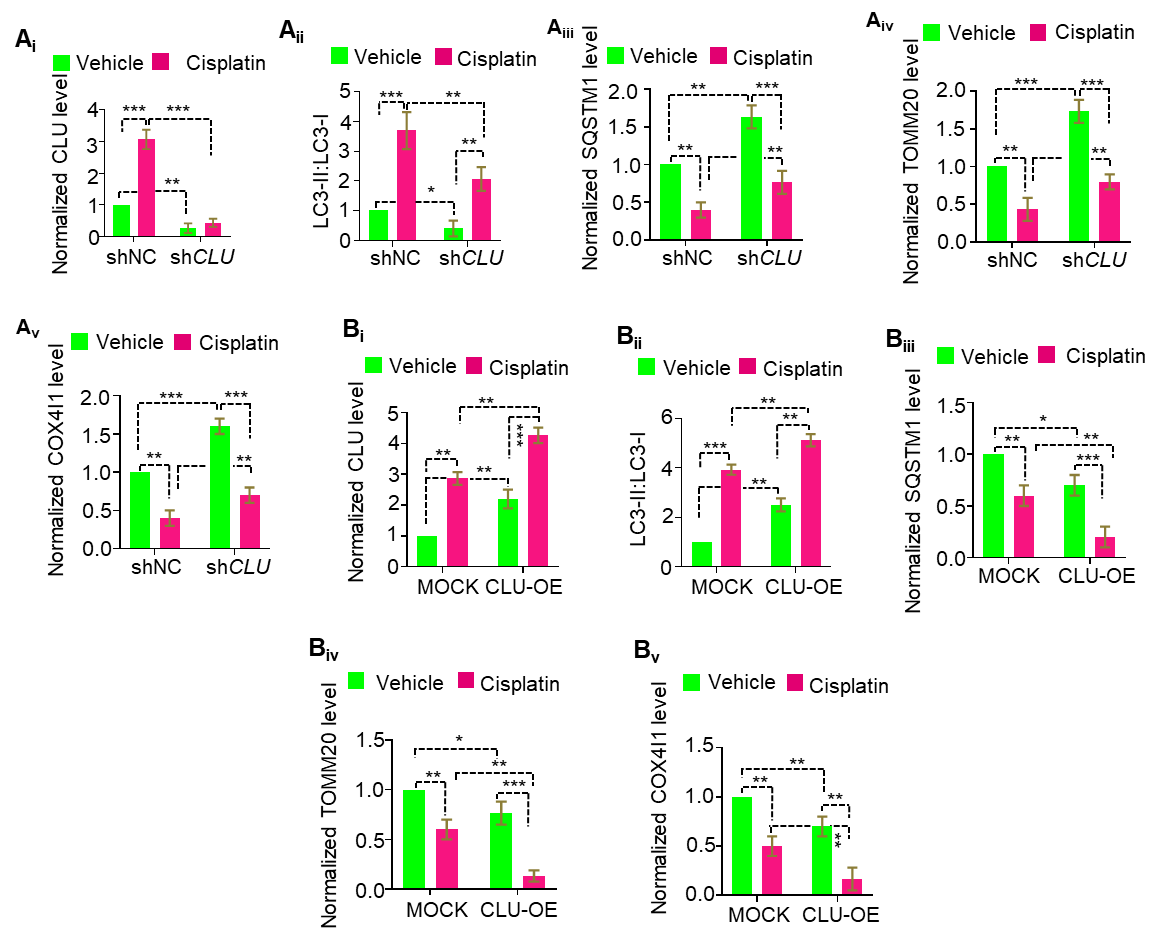


**Figure S2.** Loss and gain of CLU regulates mitophagy status in oral cancer cells during cisplatin treatment. (**A_i-v_, B_i-v_**) Densitometric analysis showing the expression of CLU, LC3, SQSTM1, TOMM20, and COX4I1 in CLU KD and CLU-OE cells treated with cisplatin (10 µM; 24 h). ACTB is a loading control and was used for fold change calculation. Data were normalized to the shNC or MOCK untreated cells (mean ± S.D., n = 3). Error bars: S.D. ∗p < 0.05, ∗∗p < 0.01, ∗∗∗p < 0.001.


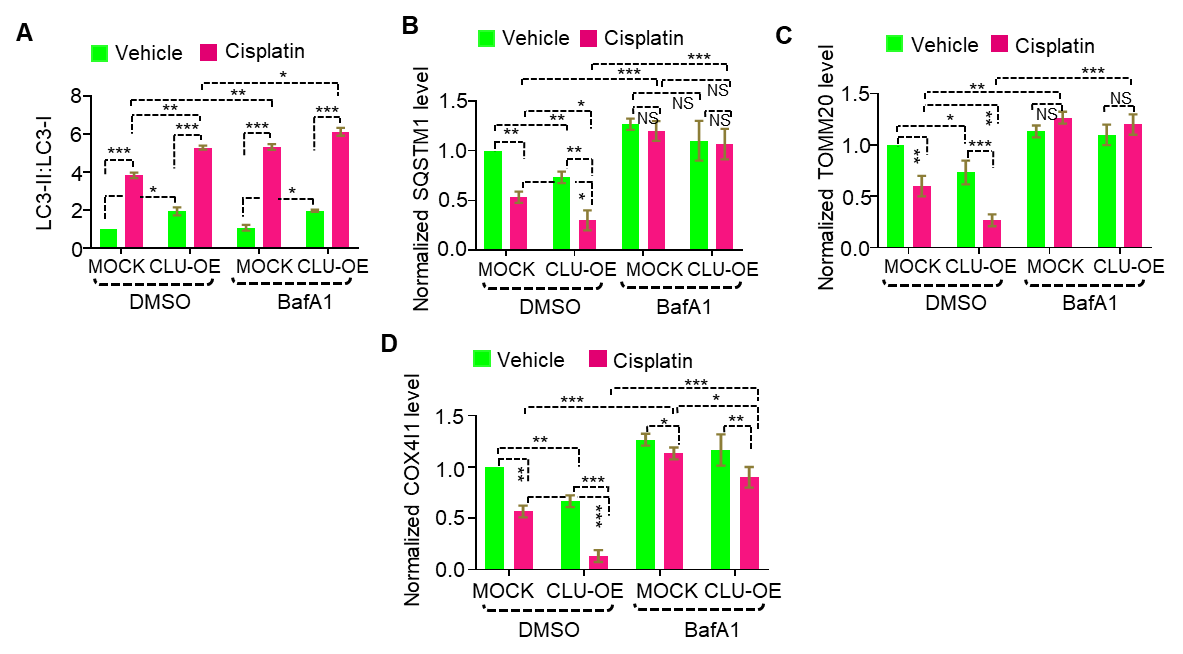


**Figure S3.** Impaired mitophagy rescued mitochondrial protein levels in CLU-OE cells in response to cisplatin treatment. (**A_i-v_, B_i-v_**) Densitometric analysis showing the expression of LC3, SQSTM1, TOMM20, and COX4I1 in CLU-OE cells treated with cisplatin (10 µM; 24 h) in combination with bafilomycin A_1_ (50 nm; 3h). ACTB is a loading control and was used for fold change calculation. Data were normalized to the MOCK untreated cells (mean ± S.D., n = 3). Error bars: S.D. ∗p < 0.05, ∗∗p < 0.01, ∗∗∗p < 0.001.


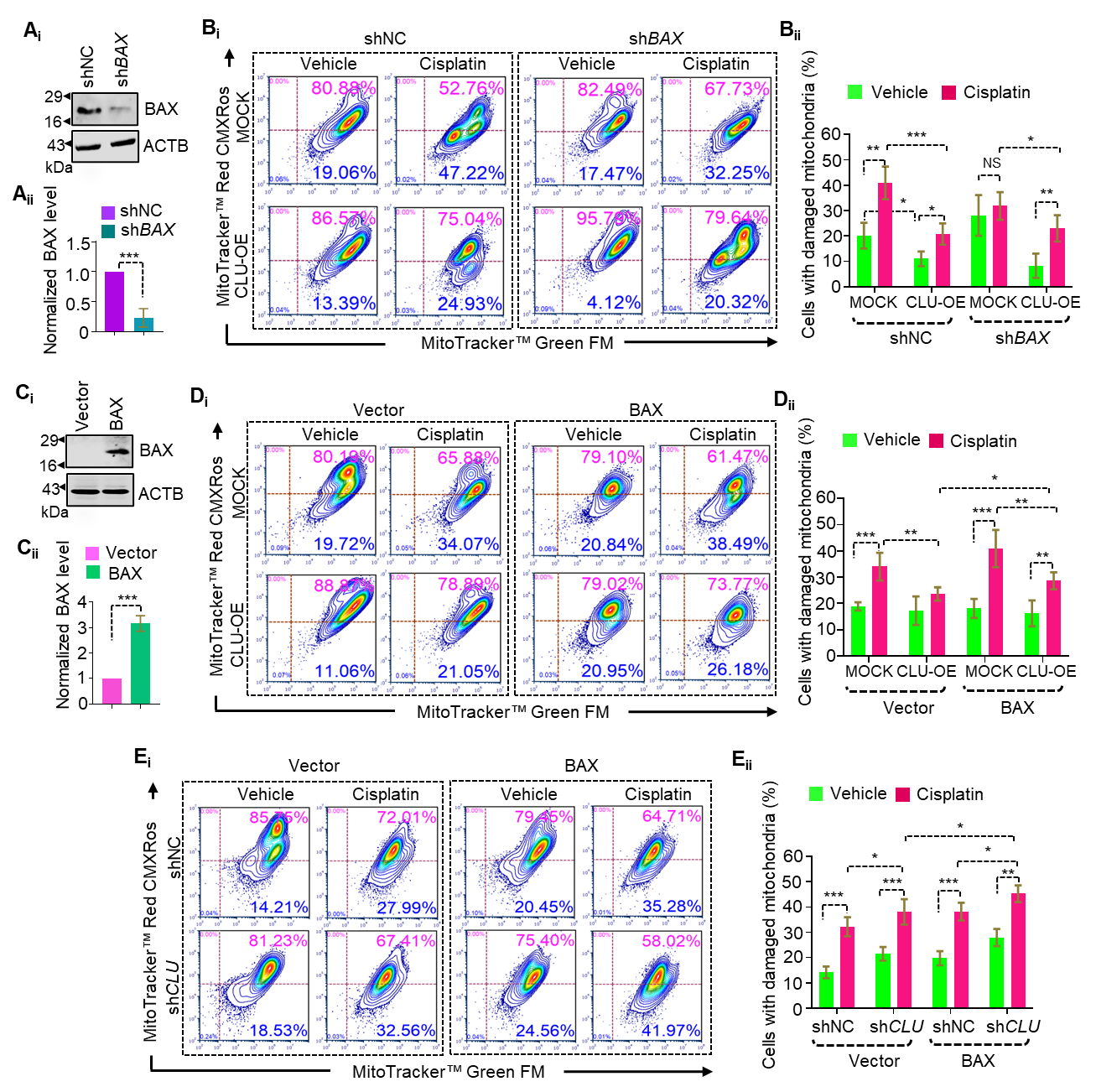


**Figure S4.** CLU is important to counter the BAX effect on mitochondrial functionality during cisplatin treatment. (**A, C**) Western blot analysis shows BAX expression during loss and gain of function in FaDu-CLU and DU 145-CLU cells, respectively. (**B_i_, D_i_**) FaDu, and DU 145 cells expressing MOCK and CLU were transiently transfected with shNC or sh*BAX*, vector or *BAX*. (**E_i_**) CAL-33 cells expressing shNC and sh*CLU* were transiently transfected with vector or *BAX* and subjected to flow cytometry analysis using MTG and MTR to assess damaged mitochondrial status during cisplatin treatment (10 µM; 24 h). (**B_ii_, D_ii_, E_ii_**) Quantification shows the cells with damaged mitochondria (%). Data were normalized to the vehicle groups, MOCK or shNC cells (mean ± S.D., n = 3). ∗p < 0.05, ∗∗p < 0.01, and ∗∗∗p < 0.001.


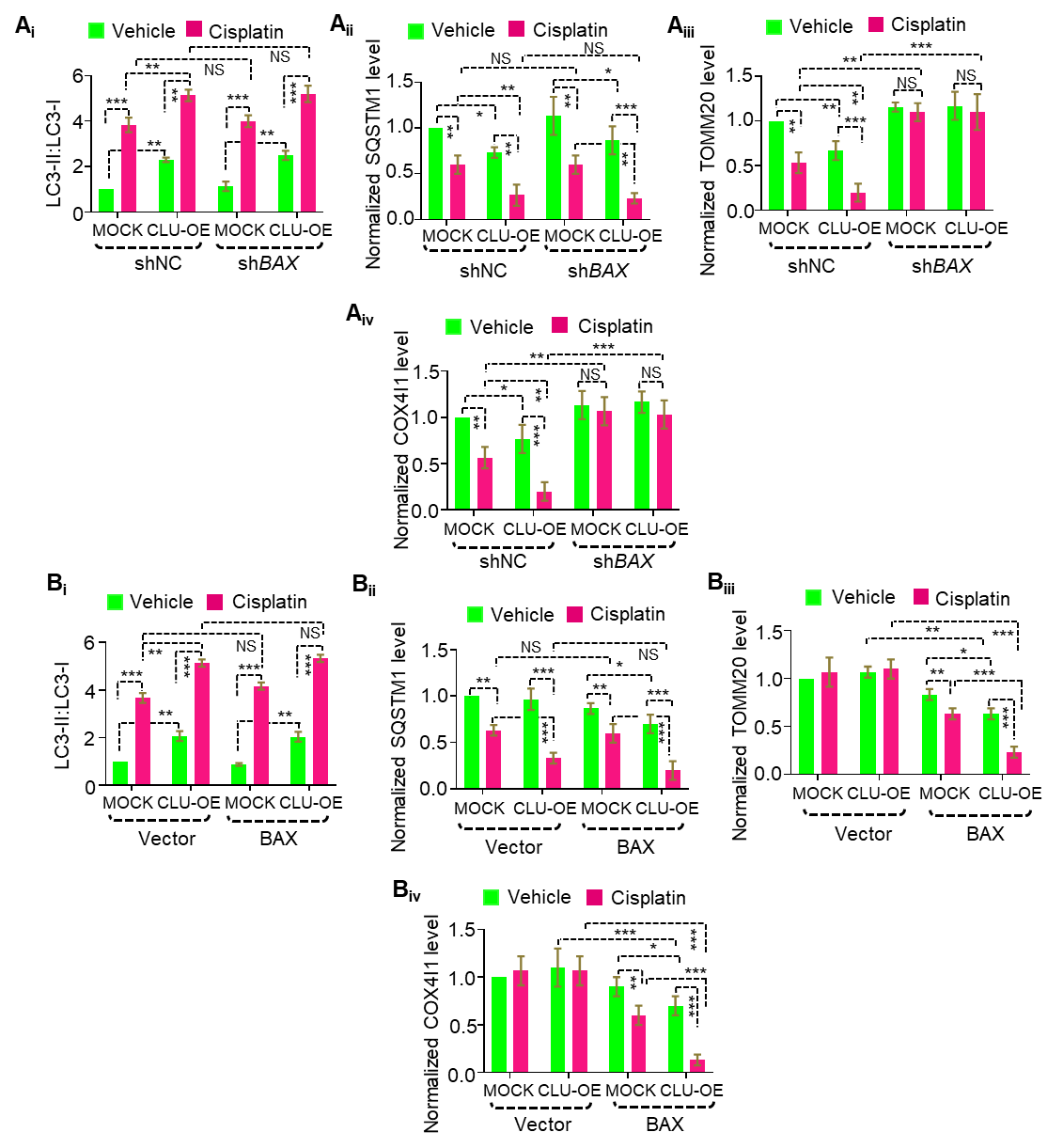


**Figure S5.** BAX is necessary for CLU-mediated clearance of damaged mitochondria generated during cisplatin treatment. (**A_i-v_, B_i-v_**) Densitometric analysis showing the expression of CLU, LC3, SQSTM1, TOMM20, and COX4I1 in CLU-OE and CLU KD cells treated with cisplatin (10 µM; 24 h). ACTB is a loading control and was used for fold change calculation. All the reported data (mean ± S.D.) were from three independent sets of the experiment with p-values (*p-value < 0.05; **p-value < 0.01; ***p-value < 0.001) considered to be statistically significant.


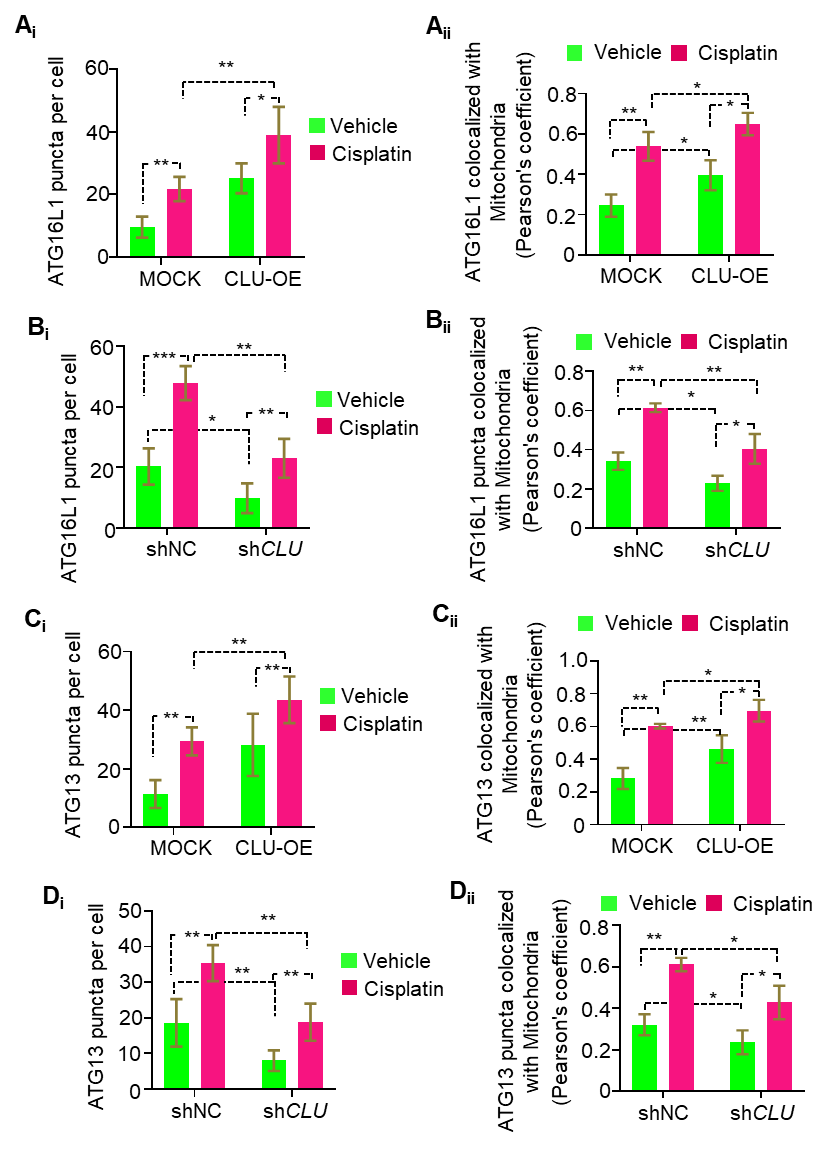


**Figure S6.** CLU promotes ATG16L1 and ATG13 puncta accumulation around mitochondria during cisplatin treatment. (**A_i_, B_i_, C_i_, D_i_**) Quantification showing the ATG16L1 and ATG13 puncta number and (**A_ii_, B_ii_, C_ii_, D_ii_**) their colocalization with TOMM20 in MOCK or CLU-OE and shNC or sh*CLU* cells treated with cisplatin (10 µM; 24h). All the reported data (mean ± S.D.) were from images taken from three independent sets of the experiment with p-values (*p-value < 0.05; **p-value < 0.01) considered to be statistically significant.


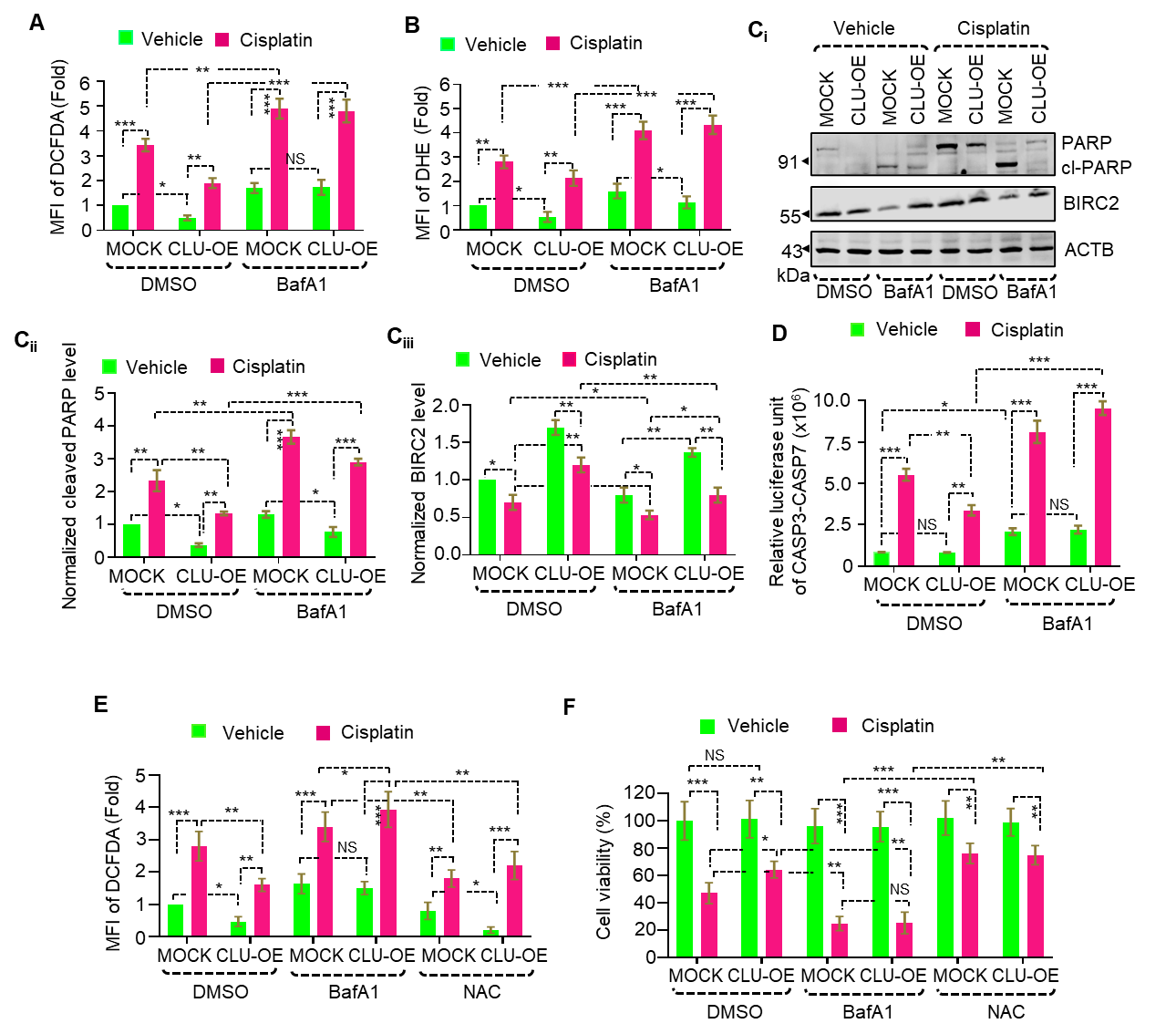


**Figure S7.** Mitophagic flux inhibition triggers ROS-dependent apoptosis. (**A, B**) Flow cytometry analysis to quantify the (**A**) peroxide levels, as indicated by DCFDA fluorescence, and (**B**) intracellular superoxide levels, as indicated by DHE fluorescence, in CLU-OE cells treated with cisplatin in combination with BafA1 (mean ± S.D., n = 3). (**C_i_**) Western blotting analysis showing the expression of cleaved PARP, and BIRC2 in CLU-OE cells treated with cisplatin (10 µM; 24 h) in combination with BafA1 (50 nM; 3 h). ACTB is a loading control and was used for fold change calculation. (**C_ii_-_iii_**) Quantification represents the densitometric analysis of fold change in cleaved PARP, and BIRC2 expression. (**D**) CASP3-CASP7 dual luciferase assay to estimate the relative expression of caspases in both MOCK and CLU-OE cells treated with cisplatin combined with BafA1 (mean ± S.D., n = 3). Error bars: S.D. ∗p < 0.05, ∗∗p < 0.01, ∗∗∗p < 0.001. (**E**) Flow cytometry analysis for DCFDA fluorescence in N-acetyl cysteine (NAC; 10 mM; 3 h)-pretreated CLU-OE cells treated with cisplatin in combination with BafA1 (mean ± S.D., n = 3). (**F**) Cell viability assay to estimate growth rate in NAC-pretreated CLU-OE cells treated with cisplatin (10 µM, 24 h) combined with BafA1. Data were normalized to the DMSO (0.1%)-treated MOCK cells (mean ± S.D., n = 5). Error bars: S.D. ∗p < 0.05, ∗∗p < 0.01, ∗∗∗p < 0.001.


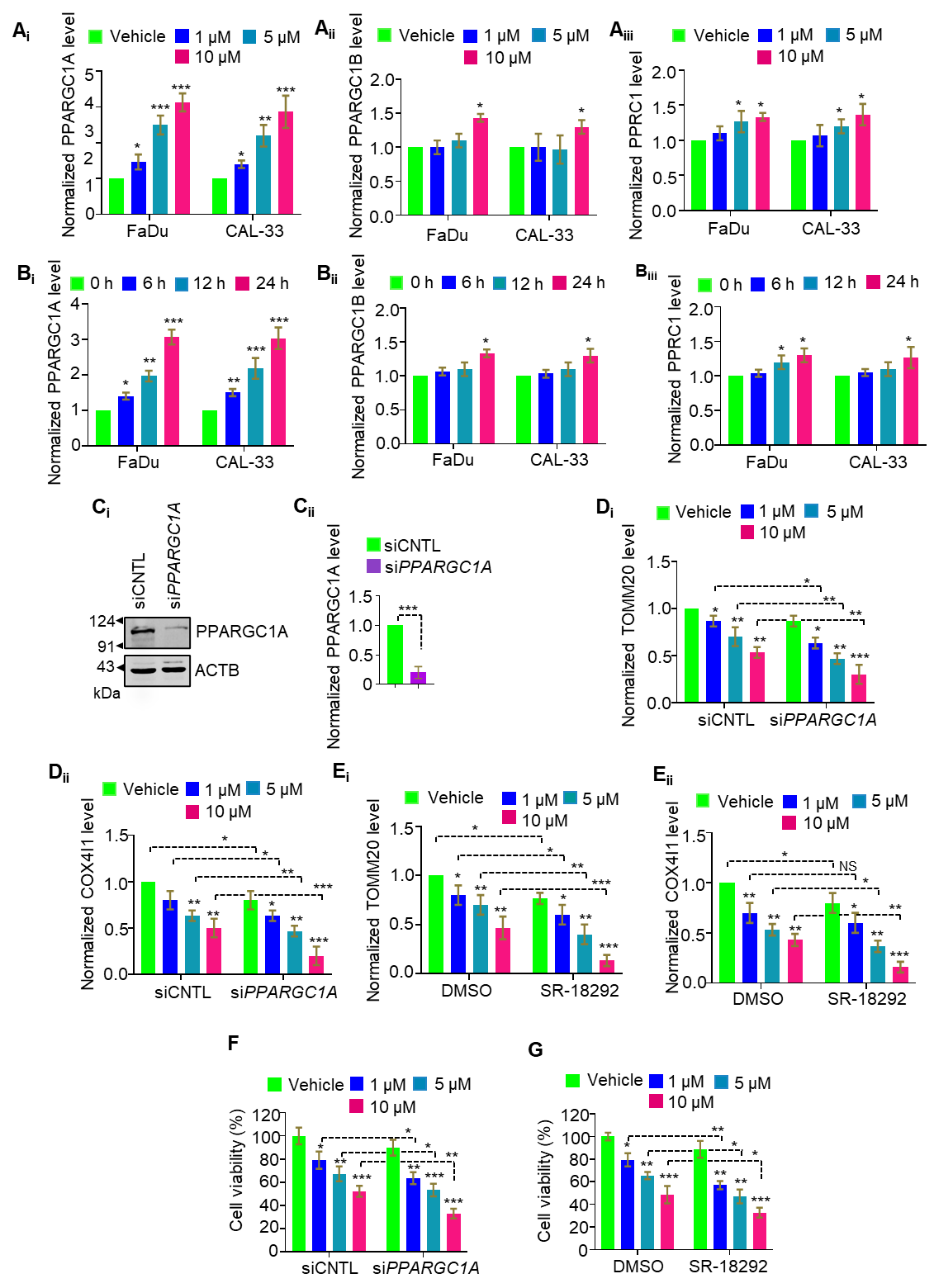


**Figure S8.** PPARGC1A inhibition triggers excessive loss of mitochondrial protein through mitophagy activation. (**A_i-iii_, B_i-iii_**) Densitometric analysis showing the expression of PPARGC1A, PPARGC1B, and PPRC1 in cisplatin-treated (1, 5, and 10 µM, 24 h; and 6, 12, and 24 h; 10 µM) FaDu and CAL-33 cells. ACTB is a loading control and was used for fold change calculation. (**C_i-ii_**) Western blotting analysis shows the expression of PPARGC1A in siCNTL and si*PPARGC1A*-transfected cells, followed by densitometry analysis using ACTB as a loading control to estimate the fold change. (**D_i-ii_, E_i-ii_**) Densitometric analysis showing the expression of TOMM20, and COX4I1 in PPARGC1A-deficient (si*PPARGC1A* and SR-18292 treated) cells treated with cisplatin (1, 5, and 10 µM, 24 h). ACTB is a loading control and was used for fold change calculation. (**F, G**) Cell viability assay in PPARGC1A-deficient (si*PPARGC1A* and SR-18292 treated) cells treated with cisplatin (1, 5, and 10 µM, 24 h). Data were normalized to the DMSO (0.1%)-treated or siCNTL-expressing MOCK cells (mean ± S.D., n = 5). Error bars: S.D. ∗p < 0.05, ∗∗p < 0.01, ∗∗∗p < 0.001.


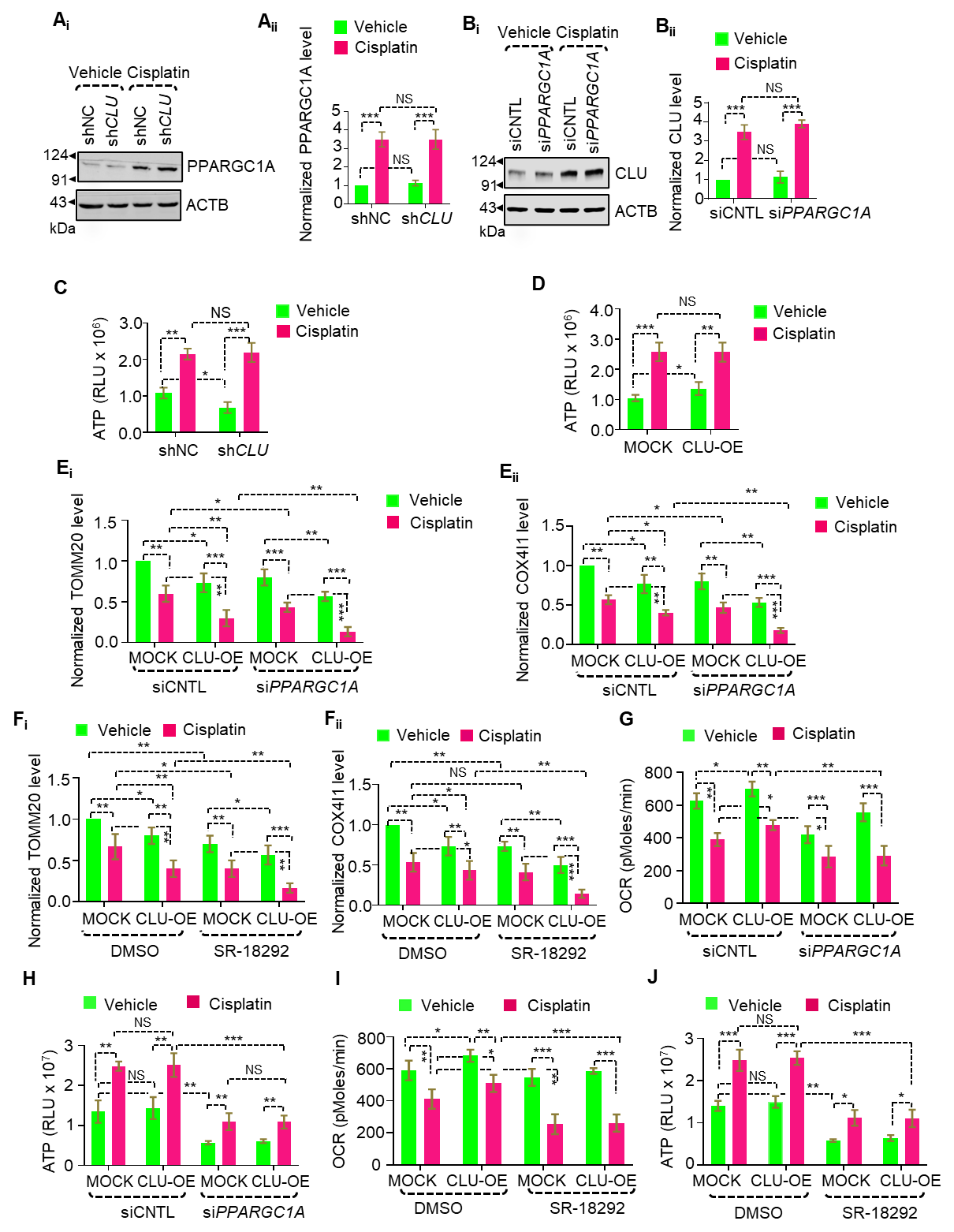


**Figure S9.** Cisplatin treatment triggers PPARGC1A-mediated mitochondrial biogenesis irrespective of CLU presence. Western blotting analysis showing the expression of (**A_i_**) PPARGC1A in shNC or sh*CLU* cells, or (**B_i_**) CLU in siCNTL- or siPPARGC1A-transfected cells treated with cisplatin (10 µM; 24 h). (**A_ii_, B_ii_**) Densitometric analysis showing the fold change of expression for PPARGC1A and CLU in CLU KD and PPARGC1A KD cells treated with cisplatin. ACTB is a loading control and was used for fold change calculation. (**C, D**) ATP measurement in shNC or sh*CLU* and MOCK or CLU-OE cells treated with cisplatin (10 µM; 24 h). RLU, relative light units. (**E_i-ii_, F_i-ii_**) Densitometric analysis showing the expression of TOMM20, and COX4I1 in PPARGC1A-deficient (si*PPARGC1A* and SR-18292 treated) MOCK or CLU-OE cells treated with cisplatin (10 µM; 24 h). ACTB is a loading control and was used for fold change calculation. (**G, I**) OCR analysis on PPARGC1A-deficient (si*PPARGC1A* and SR-18292 treated) MOCK or CLU-OE cells treated with cisplatin (10 µM; 24 h). (**H, J**) ATP measurement in PPARGC1A-deficient (si*PPARGC1A* and SR-18292 treated) MOCK or CLU-OE cells treated with cisplatin (10 µM; 24 h). RLU, relative light units. Data were normalized to the DMSO (0.1%)-treated or siCNTL-expressing MOCK cells (mean ± S.D., n = 5). Error bars: S.D. ∗p < 0.05, ∗∗p < 0.01, ∗∗∗p < 0.001.


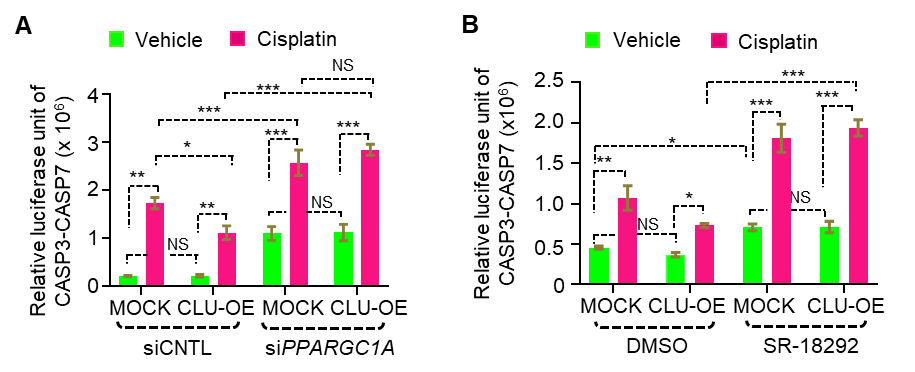


**Figure S10.** PPARGC1A inhibition activates CASP3-CASP7 activity in CLU-overexpressing cells. (**A, B**) CASP3-CASP7 dual luciferase assay estimates the relative expression of caspases in PPARGC1A-deficient (si*PPARGC1A* and SR-18292 treated) MOCK or CLU-OE cells treated with cisplatin (10 µM; 24 h). Error bars: (mean ± S.D., n = 3) ∗∗p < 0.05, ∗∗p < 0.01, and ∗∗∗p < 0.001.
